# Corticotroph isolation from *Pomc‐*eGFP mice reveals sustained transcriptional dysregulation characterising a mouse model of glucocorticoid‐induced suppression of the hypothalamus–pituitary–adrenal axis

**DOI:** 10.1111/jne.13165

**Published:** 2022-07-14

**Authors:** Peter J. Duncan, Heather McClafferty, Oscar Nolan, Qinghui Ding, Natalie Z. M. Homer, Paul Le Tissier, Brian R. Walker, Michael J. Shipston, Nicola Romanò, Thomas J. G. Chambers

**Affiliations:** ^1^ Centre for Discovery Brain Sciences University of Edinburgh Edinburgh UK; ^2^ Centre for Cardiovascular Science University of Edinburgh, Queen's Medical Research Institute Edinburgh UK; ^3^ Translational & Clinical Research Institute Newcastle University Newcastle upon Tyne UK; ^4^ Edinburgh Centre for Endocrinology and Diabetes NHS Lothian, Metabolic Unit, Western General Hospital Edinburgh UK

**Keywords:** chronic, glucocorticoid, HPA axis, recovery

## Abstract

Glucocorticoids (GC) are prescribed for periods > 3 months to 1%–3% of the UK population; 10%–50% of these patients develop hypothalamus‐pituitary–adrenal (HPA) axis suppression, which may last over 6 months and is associated with morbidity and mortality. Recovery of the pituitary and hypothalamus is necessary for recovery of adrenal function. We developed a mouse model of dexamethasone (DEX)‐induced HPA axis dysfunction aiming to further explore recovery in the pituitary. Adult male wild‐type C57BL6/J or *Pomc*‐eGFP transgenic mice were randomly assigned to receive DEX (approximately 0.4 mg kg^–1^ bodyweight day^–1^) or vehicle via drinking water for 4 weeks following which treatment was withdrawn and tissues were harvested after another 0, 1, and 4 weeks. Corticotrophs were isolated from *Pomc*‐eGFP pituitaries using fluorescence‐activated cell sorting, and RNA extracted for RNA‐sequencing. DEX treatment suppressed corticosterone production, which remained partially suppressed at least 1 week following DEX withdrawal. In the adrenal, *Hsd3b2*, *Cyp11a1*, and *Mc2r* mRNA levels were significantly reduced at time 0, with *Mc2r* and *Cyp11a1* remaining reduced 1 week following DEX withdrawal. The corticotroph transcriptome was modified by DEX treatment, with some differences between groups persisting 4 weeks following withdrawal. No genes supressed by DEX exhibited ongoing attenuation 1 and 4 weeks following withdrawal, whereas only two genes were upregulated and remained so following withdrawal. A pattern of rebound at 1 and 4 weeks was observed in 14 genes that increased following suppression, and in six genes that were reduced by DEX and then increased. Chronic GC treatment may induce persistent changes in the pituitary that may influence future response to GC treatment or stress.

## Graphical abstract

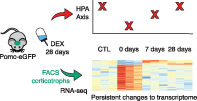

## 1 INTRODUCTION

Subsequent to the observation of the profound effect of Kendall's compound E (cortisone) administration on rheumatoid arthritis,^1^ glucocorticoids (GCs) have become a principal treatment for inflammatory conditions across most body systems. GCs are now prescribed to 1%–3% of the adult population.^2^, ^3^, ^4^ Despite the evolution of steroid‐sparing treatments, prescription rates continue to increase each year.^2^, ^4^

Exogenous GCs suppress endogenous GC production. When treatment is stopped, homeostasis should re‐activate the hypothalamus‐pituitary–adrenal (HPA) axis; however, this does not always occur, sometimes with fatal consequences.^5^ Failure of GC production following withdrawal of chronic (> 3 month) GC treatment is common (30%–50% of people immediately after stopping treatment, 10%–20% of people 6 months later^6^, ^7^, ^8^). Such cases likely have a significant clinical impact; there is a two‐ to three‐fold increase in hospital presentations with symptoms and signs of adrenal insufficiency (hypotension, hypovolaemia, cardiovascular collapse, and hypoglycaemia) in the 4‐week period following discontinuation of chronic systemic GC treatment.^9^

We are unable to accurately identify those patients at most clinical risk; meta‐analyses suggest that age, dose, and cumulative dose are likely predictors^6^, ^7^, ^9^ although these only account for some of the variability observed between patients. There is also currently no means of predicting recovery of the HPA axis at the point of starting GC therapy, and thus expensive and involved testing or increased use of steroid‐sparing agents cannot be tailored to those most at risk of adrenal insufficiency following GC withdrawal. There would be huge clinical benefits as a result of improving both the identification of those most at risk and the rate of recovery in those with a suppressed axis.

Chronic GC treatment suppresses all levels of the HPA axis. For example, there are blunted adrenocorticotrophic hormone (ACTH) and cortisol responses to insulin‐induced hypoglycaemia,^10^ impaired reactivity of the pituitary to CRH testing^10^, ^11^ and an impaired reactivity of the adrenal to ACTH_1–24._ ^12^ Importantly, the recovery of ACTH production is essential for recovery of adrenal activity in patients both following treatment for Cushing's disease and in patients where treatment with supraphysiological exogenous GCs is withdrawn.^13^ During recovery, there is marked increase in ACTH above physiological levels that precedes recovery of adrenal function.

Recovery of pituitary corticotroph function following withdrawal of chronic GC is essential for recovery of the HPA axis.^13^, ^14^, ^15^ Having found evidence for disrupted corticotroph function persisting up to 1 week following withdrawal of GC, and given in vitro evidence for long term (120 h) transcriptional changes as a result of only a brief (24 h) GC exposure,^16^ we hypothesised that chronic GC exposure programs sustained changes in the corticotroph transcriptome. We reasoned that persistent changes to transcriptional regulators or to pathways regulating ACTH synthesis and secretion might explain the delay in corticotroph and thus HPA axis recovery. We therefore established a mouse model to understand the recovery process of the HPA axis following withdrawal of chronic GC treatment.

## 2 MATERIALS AND METHODS

### 2.1 Study approval

Studies were performed according to the Animals (Scientific Procedures) Act 1986 following specific approval from the UK Home Office (Project Licence P09E1F821), following review by the University of Edinburgh Animal Research Ethics Committee and in compliance with EU directive 2010/63/EU.

**FIGURE 1:**
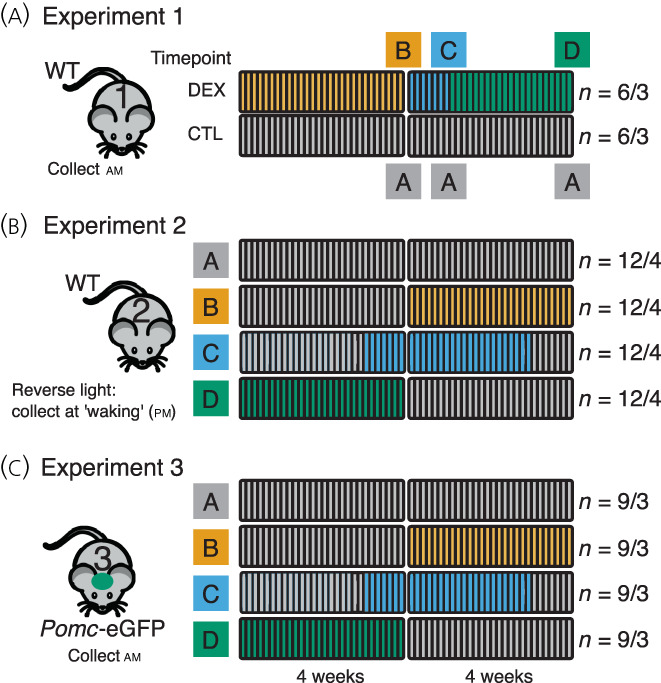
Schematic of experiments. (A) Experiment 1. C57Bl6/J mice were assigned to receive dexamethasone (DEX) in drinking water (*n* = 6 mice in three cages) or water alone (*n* = 6 mice in three cages) for 4 weeks. Control (CTL) mice received only standard drinking water (group A). Following 4 weeks treatment, two animals selected at random from each cage were sacrificed (group B). Following one further week, two further mice from each cage were collected as at time 0 (group C). The last animals were collected 4 weeks after DEX withdrawal (group D). Age matched controls were assigned to group A. WT, wild‐type. (B) Experiment 2. Here, 16 cages of three mice were randomly assigned to four treatment groups, A; receiving water in drinking water for 8 weeks, B; drinking water for 4 weeks and then DEX for 4 weeks, C; water for 3 weeks, DEX for 4 weeks then 1 week with drinking water and D; DEX for 4 weeks then drinking water for 4 weeks. All animals were collected at a single time point at the end of the experiment as above. Animals were reverse lit and collected at the point of waking. (C) Experiment 3 was conducted as Experiment 2, except that animals were not reverse lit and *Pomc*‐eGFP mice were used. Twelve cages of three animals were assigned randomly to the four groups. At the end of the experiment, tissue was collected as in Eexperiments 1 and 2, but anterior pituitary was dissected and dissociated for FACS isolation of corticotrophs. RNA from isolated corticotrophs underwent RNA‐seq. Numbers to the right refer to numbers of animals/in how many cages

Data from three experiments are presented in Figure 1. Experiment 1 aimed to confirm the safety of the 4‐week dexamethasone (DEX) exposure, assess the effect of DEX (and withdrawal) on basal corticosterone, as well as the effects on adrenal, pituitary, and hypothalamus gene expression. In Experiment 2, we aimed to assess the effect of DEX on corticosterone stimulated by waking (facilitated by ‘reverse‐lighting’ the mice). We optimised the experiment design in Experiment 2 to improve efficiency in terms of animal numbers and to remove the potential effects of age. In Experiment 3, we took advantage of the *Pomc*‐eGFP transgenic mice in order that we could directly interrogate corticotroph recovery by RNA‐sequencing (RNA‐seq).

Experiments 1 and 2 used male 8‐week‐old C57Bl6/J mice (Jackson Laboratories). Experiment 3 used male 8‐week‐old *Pomc*‐eGFP^17^ transgenic mice maintained on a C57Bl6/J background and bred in‐house. Mice were acclimatised to controlled lighting conditions in the animal facility for 2 weeks prior to each experiment, at constant temperature (22°C). Animals were fed standard chow supplemented with sunflower seeds.

### 2.2 Experiment 1

Mice (C57Bl6/J, *n* = 36) were housed six per cage (lights on 7:30 am) and then randomly assigned to receive DEX (approximately 0.4 mg^–1^ kg^–1^ day^–1^) (Sigma Aldrich) in drinking water per os, or vehicle (usual drinking water) for 4 weeks (Figure 1A). This was achieved by providing mice with drinking water supplemented with 2.51 μg ^–1^ dexamethasone phosphate (dissolved directly in water) which achieved a mean ± SD plasma concentration of 1.94 ± 2.92 nm (at start of waking period). This dose of DEX would be the allosteric equivalent of 2.5 mg for a human weighing 70 kg, which is the equivalent of approximately 16 mg of prednisolone. This ‘moderate’ dose sits in the middle of the range of a weaning dose of 40 mg down to 5 mg over 8–12 weeks commonly used to treat flares of rheumatoid arthritis or inflammatory bowel disease. Animals were weighed, and water was changed two times per week. Following 4 weeks of treatment, DEX was withdrawn, and animals were maintained in standard conditions thereafter. At withdrawal of DEX (time = 0), 1 week and 4 weeks, six animals per group, selected randomly, two per cage were sacrificed by cervical dislocation between 9:00 am and 11:00 am (early rest period). Whole pituitary, hypothalamus, and adrenal glands were dissected and frozen on dry ice and then stored at −80 °C. Adrenal glands were weighed. Trunk blood was obtained, and serum separated by centrifugation at 2000 ***g*** for 10 min at 4 °C and stored at −20 °C. For ease of comparison with Experiment 2, the three control time points have been combined for hormone assays. Hormone measurements did not vary significantly with time in the control mice (see Supporting information, Table S1A).

### 2.3 Experiment 2

Mice (C57Bl6/J, *n* = 48) were randomly assigned to four groups and were housed three animals per cage: group A (control) received drinking water for 8 weeks; group B (0 weeks recovery) received standard drinking water for 4 weeks and then DEX for 4 weeks; group C (1 week recovery) received standard drinking water for 3 weeks, then DEX for 4 weeks with a final week of recovery; group D (4 weeks recovery) receiving DEX in drinking water as above for 4 weeks with a switch to standard water for the following 4 weeks (Figure 1B). Two weeks prior to the start of the experiment, lighting was reversed (lights on 21:00 pm, lights off 09:00 am), otherwise conditions were as Experiment 1. At the end of the experiment, mice were randomly assigned to receive 30 min of restraint stress 20–60 min prior to sacrifice. Because there was no significant effect of stress on measurements taken, these two groups have been combined (see Supporting information, Table S1B). Mice were sacrificed by cervical dislocation and trunk blood collected into cold tubes prepared with 5 μL of 5% EDTA and placed on ice. Plasma was obtained by centrifugation as above and stored at −80 °C until analysis. Anterior pituitary, hypothalamus, and adrenals were dissected and frozen on dry ice and stored at −80°C. All animals were killed between 9:40 am and 11:45 am (early wake period).

### 2.4 Experiment 3

*Pomc*‐eGFP mice (*n* = 36) were randomly assigned to the four groups described in Experiment 2, at three animals per cage with lights on from 07:00 am to 19:00 pm (Figure 1C). At the end of the experimental protocol, mice were killed by cervical dislocation between 9:35 am and 10:30 am (early rest period). Plasma, hypothalamus, adrenals, and pituitaries were harvested as described above. The posterior and intermediate pituitary lobes were carefully dissected and anterior pituitaries were dissociated as reported previously.^18^ Briefly, pooled tissues, each pool from three mice, were minced with a razor blade before being placed into 2.5 mL of Dulbecco's modified Eagle's medium (DMEM) with 25 mm HEPES (LifeTechnologies) supplemented with 5 μL mL^–1^ DNAse I (Sigma) and 0.25% trypsin (Worthington). The pituitaries were digested for 20 min at 37°C with gentle shaking every 5 min. Digestion was terminated by allowing cells to settle for 5 min, removing the supernatant and trituration of the cells in 1 mL of DMEM supplemented with 50 μL of soybean trypsin inhibitor (Sigma), 50 μL of aprotinin (100 Kallikrein inhibitor units) (Sigma), and 5 μL of DNAse I. Cells were passed through a 70‐μm cell strainer (Corning) that was washed with further inhibition solution. Cells were then centrifuged for 10 min at 100 ***g*** at room temperature, the supernatant was removed, and the cells resuspended in DMEM supplemented with 4.5 g L^–1^ glucose with l‐glutamine and 25 mm HEPES (Life Technologies), 0.3% bovine serum allbumin, 1 × ITS liquid media supplement (Sigma), 4.2 μg mL^–1^ fibronectin (Sigma), and 1 × antibiotic/antimycotic solution (Sigma) (penicillin, streptomycin, and amphotericin B) and then triturated gently before being transported to the fluorescence‐activated cell sorting (FACS) facility.

### 2.5 Isolation of eGFP positive cells

Cells were resuspended in 500 μL of phosphate‐buffered saline supplemented with 25 mm HEPES and 5 mm EDTA (FACS buffer) and passed through a 35‐μm cell strainer that was washed with a further 200 μL of the FACS buffer. Draq7 was added as a vitality marker and cells sorted using a SH800 Cell Sorter (Sony). Gates were established using wild‐type pituitaries to avoid capturing eGFP negative (−ve) cells and to select single cells. Sorted single cells were resuspended into low bind Eppendorf tubes into 300 μL of Trizol and were frozen on dry ice and stored at −80 °C until sending for sequencing.

### 2.6 RNA extraction, QC, library preparation, and Illumina sequencing

RNA isolation, library preparation, and sequencing reactions were conducted at GENEWIZ, LLC. (South Plainfield, NJ, USA). Total RNA was extracted from FACS sorted cells using Qiagen RNeasy Plus Universal Mini kit in accordance with the manufacturer's instructions (Qiagen). The RNA samples were quantified using Qubit 2.0 Fluorometer (Life Technologies) and were below the limit of detection.

Ultra‐low input RNA sequencing library preparation used the SMART‐V4 kit for cDNA Synthesis (Clontech) from 10 pg to 10 ng of total RNA and polyA amplification. Illumina Nextera XT kit was used to prepare the final DNA libraries accordance with the manufacturer's instructions. Integrity of the sequencing library was assessed on an Agilent TapeStation (Agilent Technologies) and quantified by using a Qubit 2.0 Fluorometer (Invitrogen) as well as by a quantitative polymerase chain reaction (qPCR) (KAPA Biosystems).

The sequencing libraries were clustered on one lane of a patterned flowcell. After clustering, the flowcell was loaded on a HiSeq 4000 System (Illumina) or equivalent instrument in High Output Mode in accordance with the manufacturer's instructions. The samples were sequenced using a 2x150 Paired End configuration. Image analysis and base calling were conducted using the HiSeq Control Software. Raw sequence data (.bcl files) generated from the Illumina HiSeq were converted into fastq files and de‐multiplexed using bcl2fastq 2.17 software (Illumina). One mismatch was allowed for index sequence identification.

### 2.7 Steroid quantification

For Experiment 1, 11‐dehydrocorticosterone and corticosterone were measured by tandem liquid chromatography‐tandem mass spectrometry (LC‐MS/MS) in 30 μL of mouse serum at the mass spectrometry core facility, University of Edinburgh. Briefly, serum (30 μL) was enriched with internal standards (D_4_‐cortisol [D4F] and epi‐corticosterone [Epi‐B], 2.5 ng). Chloroform (10:1) was added and vortexed. The supernatant was reduced to dryness under oxygen‐free nitrogen at 60°C and reconstituted in water: acetonitrile (70 μL; 70:30 v/v). Samples were extracted alongside a calibration curve of 11‐dehydrocorticosterone and corticosterone (0.001–10 ng) with D4F and Epi‐B as internal standards (1 ng). Quantitative analysis of the extracts was carried out on a Nexera UHPLC – Sciex QTrap 6500+ LC‐MS/MS instrument, operated in positive ion mode, as described previously.^19^, ^20^

For Experiment 2, a low volume extraction method was developed for mouse plasma and combined with an LC‐MS/MS method adapted froma previous study,^21^ to detect corticosterone, 11‐dehydrocorticosterone, and DEX by LC‐MS/MS in only 10 μL of mouse plasma. Briefly, 10 μL of plasma was diluted to 100 μL with water, prepared alongside a calibration curve of 11‐dehydocorticosterone, corticosterone, and DEX. The plasma, calibration standards, and blanks were dispensed manually as aliquots (100 μL) into individual wells of a 2‐mL 96 deep‐well polypropylene plate (Waters), enriched with an internal standard solution of isotopically labelled standards (d8‐corticosterone [Cambridge isotope laboratories] and d4‐dexamethasone [Sigma‐Cerrilliant]) in methanol was added (20 μL; 10 ng). The plate was agitated and transferred to an Extrahera™ automated sample processor (Biotage) where formic acid (100 μL, 0.1%, v/v) was added to each well. Samples were incubated at room temperature (18–22 °C; 5 min) and transferred to an SLE+ 200 plate by the robot and loaded onto the SLE material under positive pressure using compressed air. The analytes were eluted from the SLE material into a deep‐well collection plate by positive pressure following the addition of dichloromethane/propan‐2‐ol (98:2; 4 × 450 μL). The eluate was reduced to dryness under a stream of heated oxygen‐free nitrogen (40°C) on an SPE Dry™ Dual Sample Concentrator System (Biotage). Once dry, extracts were dissolved in water/methanol (100 μL; 70:30 v/v), the plate was sealed with a zone‐free plate seal, and shaken on a plate shaker (10 min) before injecting directly from the 96‐well plate for LC‐MS/MS analysis.

Samples were injected (10 μL) onto a Kinetex C18 column (150 × 3 mm; 2 μm; Phenomenex) at 40°C at a flow rate of 0.5 mL min^–1^, mobile phase A – water 0.05 mm ammonium fluoride, B – methanol 0.05 mm ammonium fluoride from 55 to 100% B over 16 min, protected by a Kinetex KrudKatcher (Phenomenex). Separation was followed by analysis on a QTrap 6500+ Linear Ion Trap Quadrupole Mass Spectrometer (AB Sciex) system using a TurboIonspray source operated at 600°C. 11‐Dehydrocorticosterone, corticosterone, and DEX were detected with the following transitions: *m/z* 345.1.1➔ 121.2, *m/z* 347.1 ➔ 121.1, *m/z* 393.1 ➔ 373.2, whereas internal standards d8‐corticosterone and d4‐dexamethasone were detected with the following transitions: *m/z* 355.3 ➔ 125.1 and *m/z* 397.1 ➔ 377.2.

The peak areas of each steroid and internal standard were integrated using Quantitate in Analyst 1.6.3 (AB Sciex). Linear regression analysis of calibration standards, calculated using peak area ratios of steroid of interest to internal standard, was used to determine the concentration of the steroid in the samples. *R* ^2^ > 0.99 was considered acceptable and within each batch of samples the accuracy at the upper and lower limits were only accepted if the accuracy < 20%. The amount of steroid was calculated using linear regression analysis of the peak area ratio.

### 2.8 ACTH quantification

ACTH was measured in 25–50 μL of plasma using an ACTH ELISA kit M046006 (MD Biosciences) in accordance with the manufacturer's instructions. The assay has low cross‐reactivity with other products of *Pomc*. Samples were diluted 1:4 or 1:8. The assay had a lower limit of detection of 4 pg mL^–1^, has an intra‐assay precision (CV) of 6.7% and an inter‐assay precision (CV) of 7.1%.

### 2.9 Reverse transcription (RT)‐qPCR

RNA was extracted from tissue using the Reliaprep Miniprep System (Promega) in accordance with the standard protocol, except that treatment with DNase to remove genomic DNA was extended for 30 min. RNA purity was assessed using spectrophotometry to ensure 260/280 readings were > 1.8. RNA integrity for subsets of samples was assessed by inspection of RNA on a 1% agarose gel. RNA was reverse transcribed using Superscript IV (Thermo Fisher) in accordance with the manufacturer's instructions with random hexamers and oligo‐dT (50:50). RT‐qPCR was conducted using a StepOne Plus (Thermo Fisher) cycler and PowerSYBR (Thermo Fisher) in accordance with the manufacturer's instructions. The primers are shown in the Supporting information (Table S2) and were either Quantitect assays (Qiagen), or were designed in house and synthesised by Eurofins (UK), PCR products were assessed for size and for primer dimerisation by running 10 μL of RT‐qPCR products on a 3% agarose gel (see Supporting information, Figure S1). Relative expression was determined, with stably expressed reference genes determined using NormFinder (https://moma.dk), and the geometric mean of the best combination of two genes was used: for Experiment 1, adrenal reference genes were *Gapdh* and *Ipo8*; for pituitary, *Ppia* and *Ipo8*; and, for hypothalamus, *Ppia* and *Gapdh*. For Experiment 2 (adrenal), *Gapdh* and *Kdm2b* were used as reference genes. Data are presented as delta delta Ct versus the control; in Experiment 1, the control from the same time point and, in Experiment 2, with the control arm (group A).

### 2.10 Statistical analysis

Differences between groups were assessed by linear mixed‐effects model. Normality was assessed by visualisation of Q‐Q plots. For Experiment 1, treatment and time were used as dependent variables and cage as a random factor. For Experiments 2 and 3, group was used as the dependent variable and cage as a random factor. For assessing bodyweight, group and time were used as dependent variables and individuals within cage as random factors to allow for repeated measures. For ease of comparison of RT‐qPCR experiments and quantification of glucocorticoids, data from Experiment 1 were further analysed grouping together results from the different times in the control arm (delta Ct still relative to the time point control). This did not affect the interpretation of the results. Where a significant interaction between time and treatment was identified (Experiment 1), or an effect of group (Experiment 1, 2, and 3), pairwise comparison was made, corrected using Tukey's honestly significant difference. Statistical analysis was conducted using R, version 3.6.2 (R Foundation for Statistical Computing). *p* < 0.05 was considered statistically significant. Data are presented in box and whisker plots (with median and interquartile range) unless otherwise stated.

### 2.11 RNA‐seq bioinformatic analysis

Nextera adapters were removed from paired end FASTA files using trimgalore v0.6.5 (https://www.bioinformatics.babraham.ac.uk/projects/trim_galore/) with the flags –paired and –2colour. Quality control, including successful removal of adapters, assessment of phred scores and sequence length, were assessed using FastQC v0.11.9 (https://www.bioinformatics.babraham.ac.uk/projects/fastqc/). Trimmed reads were aligned to the mouse genome (GRCm38 release 98) using STAR v2.7.1a (https://github.com/alexdobin/STAR). Aligned reads were quantified using featureCounts v2.0.0 (https://bioconductor.org/packages/release/bioc/html/Rsubread.html) using the flags –p (paired end), −Q 20 (mapping quality >20) and –s 2 (reverse stranded).^22^ Count files were then analysed using EdgeR (https://bioconductor.org/packages/release/bioc/html/edgeR.html) keeping genes with > 0.1 counts per million (cpm) in at least three samples and with differential expression based upon trended dispersion. For comparison with qRT‐PCR data (see Supporting information, Figure S2D), cpm relative to the control group (A) was calculated for each gene. 17.5 million to 27.1 million reads were obtained from each sample of corticotrophs pooled from two‐three pituitaries (see Supporting information, Figure S3C).

## 3 RESULTS

### 3.1 Body and adrenal weight

**FIGURE 2:**
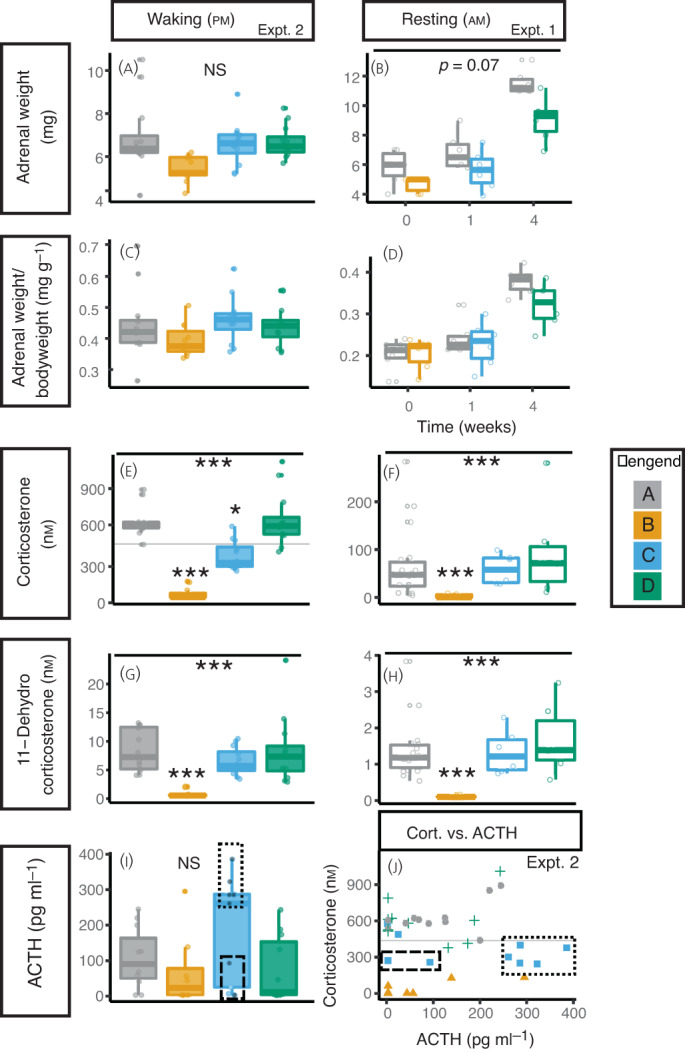
Dexamethasone reduces weight and corticosterone production that persists 1 week following treatment withdrawal. (A,B) Adrenal weight The mean weight of both adrenal glands at collection is presented with box and whisker charts from experiment 2 (A) and experiment 1 (B). (C,D) Adrenal weight/bodyweight (D,E) The adrenal weight as a proportion of bodyweight are presented for experiment 2(C) and experiment 1 (D). (E,F) Corticosterone levels. DEX treatment reduced corticosterone levels that were still reduced 1 week after stopping DEX treatment at waking (pm) (experiment 2) (E), but which had returned to normal basal levels (am; rest period) (experiment 1) (F). Corticosterone levels had returned to the level of controls 4 weeks after stopping DEX treatment. (G,H) 11‐dehydrocorticosterone levels. As (E,F). (I) ACTH levels. Data from experiment 2. The dotted box highlights mice with recovered ACTH production, the dashed box shows mice with unrecovered ACTH production; grey circles in this box are mice with Corticosterone levels below controls, blue circles are mice with corticosterone within the range of controls. (J) Relationship between plasma corticosterone and ACTH. DEX treatment (yellow triangles) reduced both plasma ACTH and corticosterone compared to controls (grey circles). One week after stopping DEX (blue squares), there was greater variation between animals with some showing high ACTH levels but lower corticosterone (dotted box) and some showing inappropriate ACTH levels for the lower corticosterone levels (dashed box). Four weeks after stopping DEX, ACTH and corticosterone levels had returned to level of controls (green crosses). Data from experiment 2. Legend. Grey dots and boxes represent control mice (Group A), yellow those who have had 4 weeks of DEX treatment (Group B), blue those one week after withdrawal of DEX (Group C) and green those 4 weeks after treatment withdrawal (Group D). Data analysed by linear mixed model with group as dependent variable and cage as random factor. Tukey‐adjusted post hoc tests compared to control group are indicated above boxes where significant differences were identified. *** p < 0.001, ** p < 0.01, * p < 0.05. *n* = 6‐12 animals from 3‐4 cages.

Weight gain was attenuated during treatment with DEX. Upon discontinuation of DEX (in group B), weight increased over 28 days to match that of control litter mates. There was a significant interaction between weight, time, and treatment *p* < 0.001 (see Supporting information, Figure S2A). Adrenal weight was not significantly reduced by DEX treatment (Figures 2A,B; see also Supporting information, Figures S2B). Adrenal/bodyweight ratio was not significantly affected by DEX or withdrawal (Figure 2C,D).

### 3.2 Steroid levels

Corticosterone levels were significantly reduced by DEX treatment (Figure 2E,F). When tested at waking (i.e., peak corticosterone levels), this finding persisted even 1 week after stopping the DEX (Figure 2F). 11‐Dehydrocorticosterone levels were also reduced by DEX but in both waking and resting phases had recovered 1 week after stopping the DEX (Figure 2G and H). ACTH levels were lower immediately following DEX, although this did not achieve statistical significance. One week after stopping the DEX, two distinct populations of animals were apparent: those with increased ACTH levels (indicating recovery of the higher HPA axis) and those with lower levels than would be expected given the relatively low corticosterone levels (Figure 2I) (Hartigan's dip test for unimodality *D* = 0.15, *p* = 0.04). Two mice had ACTH levels and corticosterone levels consistent with controls (normal corticosterone and low ACTH). This is further exemplified in Figure 2J where the animals in group B (1 week following recovery) form into disparate groups; low ACTH and low corticosterone (ongoing HPA axis suppression), high ACTH and low corticosterone (recovered pituitary, ongoing adrenal suppression), and normalised ACTH and corticosterone (recovered). DEX levels measured in the plasma from Experiment 2 were not detectable in the control group (A) or in group C or D, 1 and 4 weeks after treatment withdrawal, highlighting a carryover effect was not the reason for lower corticosterone levels in group C. Among those animals exposed to DEX (group B), plasma DEX levels reached maximally 6 nm (Figure S2C).

### 3.3 Adrenal and hypothalamic gene expression

**FIGURE 3:**
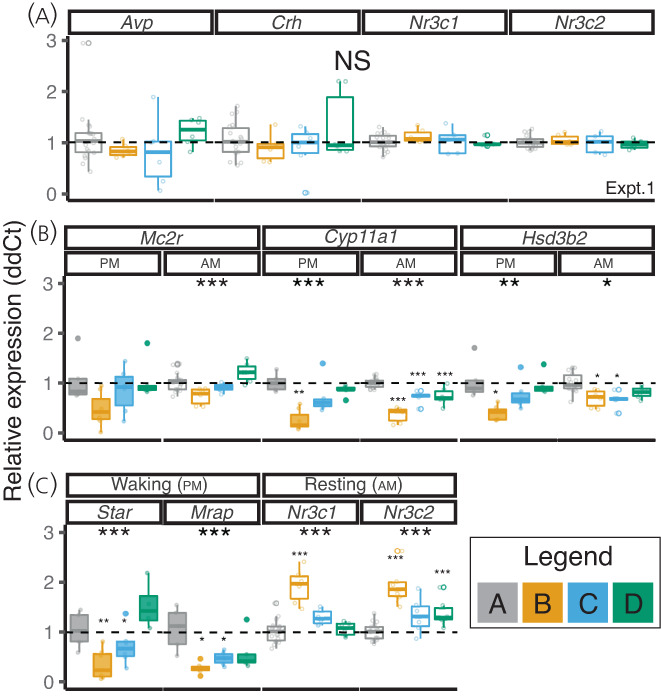
Dexamethasone (DEX) treatment induces significant and persistent changes in adrenal gene expression. (A) Hypothalamus. DEX treatment did not affect gene expression of *Avp*, *Crh*, *Nr3C1*, or *Nr3c2* in whole hypothalamus (data from Experiment 1). (B,C) Adrenal. DEX reduced the adrenal expression of the adrenocorticotrophic hormone (ACTH) receptor (*Mc2r*), steroidogenic enzymes (*Cyp11a1 Hsd3b2* and *Star*) and increased expression of the glucocorticoid receptor (*Nr3c1*) and mineralocorticoid receptor (*Nr3c2*). Data are shown from Experiment 1 (am panels) and Experiment 2 (pm panels). For ease of comparison, control samples from each time point in Experiment 1 have been combined, delta delta Ct (ddCT) values were made in comparison to time‐matched adrenal glands. Grey bars represent control mice (group A); yellow represent those who have had 4 weeks of DEX treatment (group B); blue represent those 1 week after withdrawal of DEX (group C); and green represent those 4 weeks after treatment withdrawal (group D). Data analysed by a linear mixed model with group as dependent variable and cage as random factor. Tukey‐adjusted post‐hoc tests compared to control group are indicated above bars (small asterixis) where significant differences were identified. ***p < 0.001, **p < 0.01, *p < 0.05 (*n* = 6–12 animals from three or four cages)

In Experiment 1, DEX treatment had no impact on *Avp*, *Crh*, Glucocorticoid receptor (*Nr3c1*), or Mineralocorticoid receptor *(Nr3c2*) mRNA expression in whole hypothalamus (Figure 3A).

Gene expression was examined in the adrenal glands from experiments 1 (rest period) and 2 (wake period) (Figure 3B and C). DEX treatment significantly reduced expression of mRNAs for the ACTH receptor (*Mc2r*), the steroidogenic enzymes *Cyp11a1* (side chain cleavage enzyme) and *Hsd3b2* (3‐beta hydroxylase), the first two steps in the steroidogenic pathway, *Star* the gene coding for steroid acute regulatory protein, and the gene encoding *Mrap*, which transports the ACTH receptor to the plasma membrane. One week following DEX withdrawal, there was ongoing reduced expression of *Mc2r*, *Mrap*, *Cyp11a1*, and *Star*. This was greatest in the pm/wake period when, in control animals (group A), corticosterone levels were the highest. Thus, there were altered mRNA levels in the adrenal that persisted at least 1 week after withdrawing DEX treatment, consistent with the lower corticosterone levels measured at waking in this group. mRNA levels of the glucocorticoid and mineralocorticoid receptors were significantly increased following DEX treatment, remaining significantly elevated even 4 weeks after DEX withdrawal in the case of the mineralocorticoid receptor (*Nr3c2*) (Figure 3C).

### 3.4 Pituitary gene expression

Following withdrawal of supraphysiological GC, the recovery of adrenal responsiveness to ACTH in humans follows recovery of pituitary ACTH production (presumably a result of the trophic effect of the products of POMC products/ACTH on the adrenal). Indeed, there tends to be an overshoot in ACTH production (as would be seen in primary adrenal failure) prior to recovery of the adrenal.^13^ Sustained changes to transcription of the GC responsive gene *Fkbp5* persisting 120 h beyond GC withdrawal have been found in vitro.^16^ To explore whether transcriptional dynamics were persistently affected following DEX withdrawal and might associate with HPA axis dysfunction, we used *Pomc*‐eGFP transgenic mice to isolate *Pomc* expressing cells from the anterior pituitary to assess the corticotroph transcriptome during DEX treatment and following treatment withdrawal (experiment 3). Pilot studies showed ongoing suppression of *Pomc* mRNA in isolated corticotrophs 1 week following DEX withdrawal.

**FIGURE 4:**
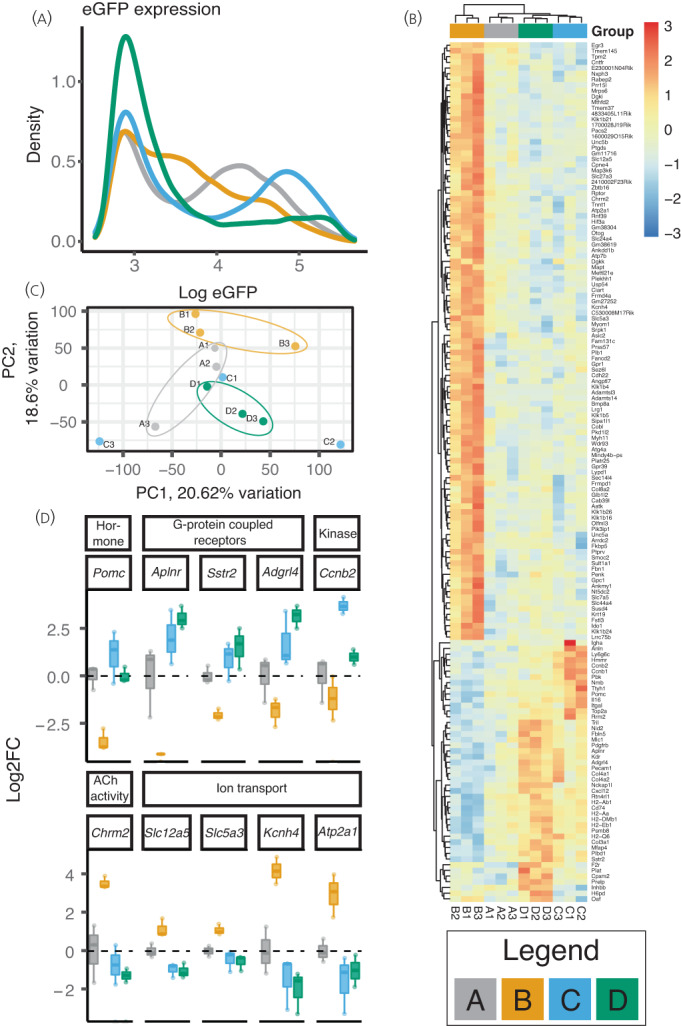
DEX treatment significantly affects the corticotroph transcriptome with changes evident 4 weeks following treatment withdrawal. (A) eGFP expression. The eGFP expression of the isolated cells is shown. In groups A (control; grey line) and C (1 week post DEX withdrawal; blue line) bimodal populations of cells are apparent. The mean fluorescence of three sorts is shown each comprising the dissociated pituitaries of 2‐3 mice. (B) Heatmap showing transcriptomic analysis of isolated cells. The 151 differentially genes with FDR <0.05 are shown. Relative expression across each row is shown as per the colour code to the right of the panel. Columns indicate each sample comprising cells isolated from 2‐3 pituitaries and are clustered according to gene expression pattern. Samples are clustered by Euclidean distance. (C) Principal component analysis (PCA) plot showing the 1st 2 principal components of PCA analysis the RNA‐seq data from corticotrophs following exposure to DEX. (D) Selected genes from RNA‐seq from classes exhibiting persisting changes following withdrawal of DEX. Log2 fold change data are shown for genes that were significantly affected by treatment and recovery along with the molecular function class to which they belong (see table 1 and 2). Data are the same as those presented in 4B. Legend. Grey lines/bars represent control mice (Group A); yellow those who have had 4 weeks of DEX treatment (Group B); blue those one week after withdrawal of DEX (Group C); and green those 4 weeks after treatment withdrawal (Group D). *n* = 3 comprising a pool of 2‐3 pituitaries.

The eGFP expression measured by FACS showed a bi‐modal distribution, suggesting the existence of two subpopulations of *Pomc* expressing cells sorted from the anterior pituitary (Figure 4A). DEX induced a shift of cells to the lower eGFP‐expressing population, which was most apparent 4 weeks after DEX withdrawal.

Treatment with DEX had no impact on the abundance of isolated cells (see Supporting information, Figure S3C), although it did induce a small but not statistically significant reduction in the proportion of GFP‐positive cells. RNA‐seq confirmed a reduction in *Pomc* mRNA in response to DEX (Figure 4B,D). Interestingly, there was variability in *Pomc* mRNA with increased *Pomc* mRNA in two pools 1 week following treatment withdrawal (group C; green), but the third pool had returned to control levels (Figure 4B and D).

Of a total of 23,247 genes (with > 0.1 read/million in more than samples), 151 were differentially expressed between the four groups (using cut off false discovery rate < 0.05). Principal component analysis showed that control, DEX treatment, and 4‐week recovery animals roughly clustered together (Figure 4C) but group C showed higher variability in both principal component (PC)1 and PC2. The 151 genes exhibiting differential expression are shown in Figure 4B. Interestingly, all four groups had a distinct transcriptomic signature, being particularly remarkable for group D despite this group showing recovery of the HPA axis by measurement of steroidogenesis. DEX treatment resulted in 63 significantly up‐ and 12 significantly down‐regulated genes (log2 fold change > 2) (Table 1). Volcano plots demonstrating differentially expressed genes across the different groups are shown in the Supporting information (Figure S4); the atypical appearance here is a result of the false discovery rate (FDR) having been determined by a comparison made across all four groups.

**TABLE 1:** One hundred and fifty‐one differentially expressed genes and the time points at which they were up‐ or down‐regulated

B\D	Up	NC	Down	D/C
Up	*Plb1*, *Krt19*	*Adamts14*, *Fancd2*, *Klk1b4*		Up
	*Fbn1*, *Ido1*, *Penk*, *Klk1b24*, *Oaf*, *Col8a2*	*Ptgds*, *Klk1b21*, *Tmem37*, *Dgki*, *Klk1b26*, *4833405L11Rik*, *Cdh22*, *Sult1a1*, *Pacs2*, *Gpc1*, *Olfml3*, *Nt5dc2*, *Prr15l*, *Atg4a*, *Gpr39*, *Lypd1*, *Arrdc2*, *Tnnt1*, *Klk1b16*, *Lrg1*, *C530008M17Rik*, *Slc44a4*, *Cpne4*, *Slc7a5*, *Fkbp5*, *Mthfd2*, *Unc5b*, *Gpr1*, *1700028J19Rik*, *Fam131c*, *Pik3ip1*, *Ttyh1*, *Ccnb1*, *Usp54*, *Plekhh1*, *Ciart*, *E230001N04Rik*, *Ankmy1*, *Glb1l2*, *Nxph3*, *Aatk*, *Cobl*, *Smoc2*, *Ankdd1b*, *Mindy4b‐ps*, *Frmd4a*, *Sez6l*, *Atp7b*, *Gm38619*, *Lrrc75b*, *Sipa1l1*, *Angptl7*, *Gm27252*, *Sec14l4*, *Platr25*, *Unc5a*, *Slc5a3*, *Cab39l*, *Mapt*, *Ptprv*, *Wdr93*, *Rabep2*, *Fstl3*, *Prss57*, *Slc27a3*, *Susd4*, *2410002F23Rik*, *Slc24a4*, *Mrps6*, *Frmpd1*	*Adamtsl3*, *Mettl21e*, *Bmp8a*, *Slc12a5*, *Myom1*, *Gm11716*, *Klk1b5*, *Myh11*, *Dgkk*, *Asic2*, *Pkd1l2*, *Gm38304*	NC
		*Atp2a1*, *Map3k6*, *Cntfr*, *Otog*	*Kcnh4*, *Hif3a*, *Tpm2*, *Rnf39*, *1600029O15Rik*, *Chrm2*	Down
NC	*Hmmr*, *Col4a1*, *Anln*, *Col4a2*	*Ttyh1*, *Ccnb1*		Up
	*Tril*, *Cpxm2*, *H6pd*, *Prelp*, *F2r*, *Fbln5*, *Plat*, *Nid2*, *Mlc1*	*Tmem145*, *Srpk1*, *Zbtb16*, *Cxcl12*, *Rptor*		NC
		*Inhbb*	*Egr3*	Down
Down	*Ly6g6c*, *Aplnr*, *Pbk*, *Top2a*, *Adgrl4*, *Il16*, *Plbd1*, *Kdr*, *Pdgfrb*, *Sstr2*, *Rrm2*, *H2‐Q6*, *Itgal*, *Pecam1*	*Nmb*, *Ccnb2*, *Pomc*	*Igha*	Up
	Col3a1, Cd74, Nckap1l, H2‐Aa, H2‐DMb1, H2‐Eb1, H2‐Ab1, Mfap4	Psmb8, Rtn4rl1		NC

*Note*: Genes were clustered into 27 subsets based upon their expression relative to controls in each treatment group vs. group B and group C in rows and group D in columns. Very few genes demonstrate persistent change in expression in the same direction over all three time points.

We used our dataset of 151 significantly changed genes (FDR < 0.05) to identify genes that showed persistent changes with log2 fold change greater than 1 vs. controls (group A). Only small groups of genes showed a persistent change in expression as a result of DEX exposure. One (*Igha*) was suppressed by DEX and was also suppressed 1 week after treatment withdrawal and eight genes were both upregulated by DEX and remained elevated 1 week after withdrawal (*Plb1*, *Krt19*, *Fbn1*, *Ido1*, *Penk*, *Klk1b24*, *Oaf*, and *Col8a2)*. Two of these remained increased 4 weeks post DEX withdrawal (*Plb1* and *Krt19*) (Table 1; see also Supporting information, Figure S4). The majority of differentially expressed genes demonstrated increased expression by DEX and then returned to baseline at 1 and 4 weeks (73 genes).

Another pattern of ‘rebound’ expression was observed (Table 1). These groups may represent genes that were directly or indirectly stimulated or repressed by GC. Fourteen genes were downregulated by DEX and then were higher at 1 or 4 weeks, and six genes were upregulated by DEX and then suppressed.

Ontological analysis using Protein Analysis Through Evolutionary Relationships, http://pantherdb.org,^23^ identified transporter activity (e.g. GO:0005215; GO:0015075) and kinase activity (GO:0016301) as the molecular processes most affected by DEX (Table 2). Dividing these genes into those up‐ or down‐regulated by DEX further highlighted some classes of molecular function of importance: genes with peptidase/hydrolase were enriched in those down‐regulated by DEX, particularly a number of Kalikrein genes and transporters (see Supporting information, Table S3). Of those suppressed by DEX, antigen binding, extra cellular matrix components, and G‐protein coupled receptors were significant (see Supporting information, Table S4).

**TABLE 2:** Over‐representation test for classes of genes with significant differential expression in Experiment 3

PANTHER GO‐Slim Molecular Function	Mus musculus ‐ REFLIST (22265)	Number of genes	Number of expected genes	Over or under represtation	A vs B (fold Enrichment)	(P‐value)	
Transporter activity (GO:0005215)	782	10	5.41	+	1.85	0.05	*Atp7b Slc7a5 Ttyh1 Slc24a4 Atp2a1 Slc12a5 Slc27a3 Pkd1l2 Slc5a3 Kcnh4*
Ion transmembrane transporter activity (GO:0015075)	581	9	4.02	+	2.24	0.02	*Atp7b Slc7a5 Ttyh1 Slc24a4 Atp2a1 Slc12a5 Pkd1l2 Slc5a3 Kcnh4*
Inorganic molecular entity transmembrane transporter activity (GO:0015318)	550	9	3.8	+	2.37	0.01	*Atp7b Slc7a5 Ttyh1 Slc24a4 Atp2a1 Slc12a5 Pkd1l2 Slc5a3 Kcnh4*
Kinase activity (GO:0016301)	572	8	3.96	+	2.02	0.05	*Srpk1 Ccnb2 Dgki Dhkk Ccnb1 Pdgfrb Cab39l Map3k6*
Phosphotransferase activity, alcohol group as acceptor (GO:0016773)	534	8	3.69	+	2.17	0.03	*Srpk1 Ccnb2 Dgki Dgkk Ccnb1 Pdgfrb Cab39l Map3k6*
Metal ion transmembrane transporter activity (GO:0046873)	310	7	2.14	+	3.26	0.01	*Atp7b Slc24a4 Atp2a1 Slc12a5 Pkd1l2 Slc12a5 Kcnh4*
Cation transmembrane transporter activity (GO:0008324)	424	7	2.93	+	2.39	0.03	*Atp7b Slc24a4 Atp2a1 Slc12a5 Pkd1l2 Slc5a3 Kcnh4*
Inorganic cation transmembrane transporter activity (GO:0022890)	395	7	2.73	+	2.56	0.02	*Atp7b Slc24a4 Atp2a1 Slc12a5 Pkd1l2 Slc5a3 Kcnh4*
Active transmembrane transporter activity (GO:0022804)	218	5	1.51	+	3.32	0.02	*Atp7b Slc24a4 Atp2a1 Slc12a5 Slc5a3*
Receptor regulator activity (GO:0030545)	255	5	1.76	+	2.83	0.03	*Lypd1 Il16 Inhbb Bmp8a Dkk3*
Monovalent inorganic cation transmembrane transporter activity (GO:0015077)	261	5	1.81	+	2.77	0.04	*Slc24a4 Atp2a1 Slc12a5 Slc5a3 Kcnh4*
Active ion transmembrane transporter activity (GO:0022853)	156	5	1.08	+	4.63	<0.01	*Atp7b Slc24a4 Atp2a1 Slc12a5 Slc5a3*
Organic cyclic compound binding (GO:0097159)	1680	4	11.62	−	0.34	0.01	*Chrm Ciart Mrps6 Gata2*
Kinase binding (GO:0019900)	158	4	1.09	+	3.66	0.02	*Ccnb2 Nell2 Myom1 Ccnb1*
Heterocyclic compound binding (GO:1901363)	1638	4	11.33	−	0.35	0.01	*Chrm2 Ciart Mrps6 Gata2*
Calcium ion transmembrane transporter activity (GO:0015085)	84	3	0.58	+	5.16	0.02	*Slc24a4 Atp2a1 Pkd1l2*
cytokine activity (GO:0005125)	116	3	0.8	+	3.74	0.05	*Il16 Inhbb Bmp8a*
Nucleic acid binding (GO:0003676)	1293	3	8.94	−	0.34	0.02	*Ciart Mrps6 Gata2*
Carboxypeptidase activity (GO:0004180)	30	2	0.21	+	9.64	0.02	*Cpxm2 Mindy4b‐ps*
Postsynaptic neurotransmitter receptor activity (GO:0098960)	49	2	0.34	+	5.9	0.05	*Chrm2 Lypd1*
Growth factor binding (GO:0019838)	38	2	0.26	+	7.61	0.03	*Gpc1*
Glycosaminoglycan binding (GO:0005539)	42	2	0.29	+	6.88	0.03	*Nell2 Rtn4rl1*
Acetylcholine receptor activity (GO:0015464)	28	2	0.19	+	10.33	0.02	*Chrm2 Lypd1*
Cyclin‐dependent protein serine/threonine kinase regulator activity (GO:0016538)	35	2	0.24	+	8.26	0.02	*Ccnb1 Ccnb2*
Acetylcholine binding (GO:0042166)	28	2	0.19	+	10.33	0.02	*Chrm2 Lypd1*
Neuropeptide binding (GO:0042923)	21	2	0.15	+	13.77	0.01	*Sstr1 Gpr1*
Heparin binding (GO:0008201)	27	2	0.19	+	10.71	0.02	*Nell2 Rtn4rl1*
Fibroblast growth factor binding (GO:0017134)	6	1	0.04	+	24.1	0.04	*Gpc1*
Lipopolysaccharide binding (GO:0001530)	7	1	0.05	+	20.65	0.05	*Tril*

*Note*: Genes with differential expression were assessed for over‐enrichment using PANTHER, version 15.0, GO‐slim molecular function. Binomial test with Bonferroni correction was applied to compare the list of differentially expressed genes with that expected.

Further analysis examining the molecular function of pathways that were changed across all groups is shown in Table 3. Here, pathways that were identified as significantly up‐ or down‐regulated in comparisons between control and each of the three treatment groups are shown. For example, G protein‐coupled receptor activity (GO:0004930) was down‐regulated by DEX but showed a rebound increase. Cation transmembrane transporter activity (GO:0008324) on the other hand was up‐regulated by DEX and then reduced during the recovery period.

**TABLE 3:** Over‐representation test for classes of genes with changes across three groups with direction of change in expression

Molecular function	Mus Mus genes	Enriched	Expected	Fold enrichment	Significance	A vs B	A vs C	A vs D	Genes
G protein‐coupled receptor activity (GO:0004930)	405	3	0.56	5.32	0.02	Down	Up	Up	*Aplnr; Sstr2; Adgrl4*
Neuropeptide binding (GO:0042923)	21	1	0.03	34.2	0.03	Down	Up	Up	*Sstr2*
Peptide binding (GO:0042277)	179	2	0.25	8.02	0.03	Down	Up	Up	*Sstr2; Aplnr*
Amide binding (GO:0033218)	194	2	0.27	7.4	0.03	Down	Up		*Aplnr; Sstr2*
Cyclin‐dependent protein serine/threonine kinase regulator activity (GO:0016538)	35	1	0.05	20.52	0.05	Down	Up		*Ccnb2*
Molecular transducer activity (GO:0060089)	1349	5	1.88	2.66	0.04	Down	Up		*Aplnr; Pdgfrb; Ccnb2; Sstr2; Adgrl4*
Rac GTPase binding (GO:0048365)	26	1	0.04	27.62	0.04	Down		Up	*Nck1l*
Acetylcholine binding (GO:0042166)	28	2	0.12	17.1	<0.01	Up	Down	Down	*Lypd1; Chrm2*
Acetylcholine receptor activity (GO:0015464)	28	2	0.12	17.1	0.007	Up	Down	Down	*Lypd1; Chrm2*
Actin filament binding (GO:0051015)	84	2	0.35	5.7	0.05	Up	Down	Down	*Myom1; Tpm2*
Cation transmembrane transporter activity (GO:0008324)	424	7	1.77	3.95	<0.01	Up	Down	Down	*Slc12a5; Slc5a3; Atp7b; Slc24a4; Kcnh4; Atp2a1; Pkd1l2*
Hormone binding (GO:0042562)	52	2	0.22	9.21	0.02	Up	Down	Down	*Lypd1; Chrm2*
Inorganic cation transmembrane transporter activity (GO:0022890)	395	7	1.65	4.24	<0.01	Up	Down	Down	*Slc12a5; Slc5a3; Atp7b; Slc24a4; Kcnh4; Atp2a1; Pkd1l2*
Inorganic molecular entity transmembrane transporter activity (GO:0015318)	550	8	2.3	3.48	<0.01	Up	Down	Down	*Slc12a5; Slc5a3; Atp7b; Slc24a4; Kcnh4; Slc7a5; Atp2a1; Pkd1l2*
Ion transmembrane transporter activity (GO:0015075)	581	8	2.43	3.3	<0.01	Up	Down	Down	*Slc12a5; Slc5a3; Atp7b; Slc24a4; Kcnh4; Slc7a5; Atp2a1; Pkd1l2*
Metal ion transmembrane transporter activity (GO:0046873)	310	7	1.29	5.41	<0.001	Up	Down	Down	*Slc12a5; Slc5a3; Atp7b; Slc24a4; Kcnh4; Atp2a1; Pkd1l2*
Monovalent inorganic cation transmembrane transporter activity (GO:0015077)	261	5	1.09	4.59	<0.01	Up	Down	Down	*Scl12a5; Slc5a3; Kcnh4; Atp2a1*
Postsynaptic neurotransmitter receptor activity (GO:0098960)	49	2	0.2	9.77	0.02	Up	Down	Down	*Lypd1; Chrm2*
Transmembrane transporter activity (GO:0022857)	701	8	2.93	2.73	<0.01	Up	Down	Down	*Slc12a5; Slc5a3; Atp7b; Slc24a4; Kcnh4; Slc7a5; Atp2a1; Pkd1l2*
Ammonium ion binding (GO:0070405)	80	2	0.33	5.99	0.05	Up	Down		*Lypd1; Chrm2*
Calcium ion transmembrane transporter activity (GO:0015085)	84	3	0.35	8.55	<0.01	Up	Down		*Slc24a4; Atp2a1; Pkd1l2*
Potassium ion transmembrane transporter activity (GO:0015079)	139	3	0.58	5.17	0.02	Up		Down	*Slc12a5; Kcnh4*
Transporter activity (GO:0005215)	782	9	3.27	2.76	<0.01	Up		Down	*Slc12a5; Kcnh4; Pkd1l2*
Extracellular matrix structural constituent (GO:0005201)	11	1	0.05	21.76	0.05	Up		Up	*Fbn1*
Oxidoreductase activity, acting on single donors with incorporation of molecular oxygen, incorporation of two atoms of oxygen (GO:0016702)	10	1	0.04	23.94	0.04	Up		Up	*Ido1*
DNA‐binding transcription factor activity (GO:0003700)	618	2	0.31	6.55	0.04		Down	Down	*Hif3a; Egr3*

*Note*: Genes with differential expression were assessed for over‐enrichment using PANTHER version 15.0, GO‐slim molecular function. Binomial test with Bonferroni correction was applied to compare the list of differentially expressed genes with that expected. Data are shown from classes identified in more than one comparison.

We assessed differentially expressed gene sets for regulator regions using I‐cis.^24^ Consistent with induction by GC, we found enrichment for genes associated with binding sites for *Nr3c1* (the glucocorticoid receptor) (normalised enrichment score 5.48, > 3 considered significant). Interestingly, we did not find enrichment for GR in the genes suppressed by DEX; this might be because the algorithm is less effective at identifying the distal enhancers associated with DEX suppression,^25^ or because the suppressive mechanism may depend on intermediary transcription factors.^16^, ^26^

## 4 DISCUSSION

We exposed mice to the synthetic GR agonist, DEX for 4 weeks in drinking water and show that sustained suppression of the HPA axis persisting for at least 1 week following treatment withdrawal. We found that, at 1 week following DEX withdrawal, some animals show a compensatory increase in plasma ACTH and corticotroph *Pomc* expression, whereas others still demonstrate suppressed corticotroph action given the relative corticosterone deficiency. DEX has a significant effect on the corticotroph transcriptome; although, 1 week following treatment withdrawal, the majority of genes affected by DEX have returned to control levels, differential expression of several genes persists at least 1 week after withdrawal of DEX.

As expected, we found evidence for reduced adrenal function persisting 1 week following withdrawal of DEX treatment.^27^, ^28^ A significant reduction in the abundance of transcripts for genes regulating steroidogenesis was found 1 week following DEX withdrawal. We did not see significant reductions in adrenal size as seen in other models of glucocorticoid exposure^27^, ^29^; this may relate to the fact that the adrenals still exhibited growth during the experimental period (Figure 2B,D), or because the dose of DEX we used was comparatively small.

The adrenal response to ACTH is reduced both in humans and in rodent models following withdrawal of chronic exogenous GC treatment.^12^, ^28^ Previous studies highlight normalised ACTH plasma measurements in the context of ongoing adrenal insufficiency, suggesting an ongoing defect at the level of the adrenal.^12^, ^28^, ^30^ However, another interpretation of these data is that ACTH levels should be increased in the milieu of relative adrenal insufficiency and thus normalised ACTH levels are suggestive of ongoing attenuated corticotroph (or hypothalamic) function. Furthermore, expression of the steroidogenic enzymes that remain suppressed 1 week following DEX withdrawal is driven by ACTH,^31^ which might also point towards ongoing impaired activity at the higher HPA axis. Taken together, our data indicate that exogenous GC exposure suppresses both adrenal and pituitary function and suggest that recovery of adrenal function is dependent (at least in part) upon recovery of the pituitary.

One week following withdrawal of DEX, we find heterogeneity in the HPA axis activity of mice. In the waking phase (pm), we show that, although five of nine mice exhibit the expected increase in ACTH associated with impaired adrenal function (consistent with recovery of corticotroph activity), two of nine have ACTH that is inappropriately low given the low corticosterone levels. We see a similar pattern in the transcriptome of isolated corticotrophs where one pool out of three shows low (similar to control) levels of *Pomc* transcript when this should be elevated in the context of adrenal insufficiency. This variability is reminiscent of human studies where only a proportion of patients are found to have ongoing adrenal insufficiency following withdrawal of exogenous GC (e.g., 10% of patients at 6 months).^7^, ^32^ The data suggest inter‐individual differences in the trajectory of recovery of the mice in terms of ACTH release that could be established in further studies looking at further ‘snap shots’ of the recovery process following DEX withdrawal.

A number of possibilities could account for these differences: (1) we show variance in the levels of plasma DEX attained as a result of the exposure in drinking water, thus the variability could be attributed to different dose exposures as a result of differences in water intake as mice were co‐housed; (2) social hierarchy may affect HPA axis activity as observed in *Cynomolgus* ^33^ (although we pooled mice from the same cage for the RNA‐seq experiments); and (3) finally, factors that influence GC sensitivity of the HPA axis, for example epigenetic changes in the hypothalamus as a result of early life or prenatal stress exposure,^34^, ^35^, ^36^ could account for inter‐individual differences.

DEX at 0.4 mg kg^–1^ likely reaches lower concentrations within the blood brain barrier than plasma; 0.2 mg kg^–1^ mouse^–1^ reached brain concentrations of 30% that of plasma as a result of extrusion by *Mdr1/Abcb1*,^37^ which helps to reduce accumulation of toxins within the brain, and exports DEX. Thus, a relative GC deficient state was likely induced by our DEX exposure. We did not find changes in whole hypothalamus transcript levels for *Crh* or *Avp* when measured in the rest period. The magnocellular component (which does not contribute directly to HPA axis activity) in which these transcripts are also found abundantly may account for the lack of change here. As we have dissected whole hypothalamus, we are unable to assess specific changes in the parvocellular neurones that regulate anterior pituitary hormone release.

We explored recovery at the level of the pituitary further by isolating corticotrophs from *Pomc*‐eGFP mice. The eGFP profile of the isolated cells exhibited a bimodal distribution, which was most apparent in control mice and those 1 week following withdrawal (group A and C); at time points 1 and 4 weeks following DEX withdrawal (groups B and D), there was a shift in the population to the left with a greater proportion of ‘low eGFP’ expressing cells.

Two possibilities to account for multiple populations of cells arise here. First, inclusion of melanotrophs that also produce large quantities of *Pomc*. These cells were removed by careful dissection of the *pars intermedia*, although some melanotrophs may be present in the anterior pituitary.^38^ We did not find any association between expression of *Pcsk2* and *Pax7*, markers of melanotrophs, and those samples with larger populations of eGFP ‘high’ cells (see Supporting information, Figure S3B). Furthermore, our own experience with dispersed GFP positive cells in vitro suggests that < 1% of cells fail to respond to CRH/AVP, a mechanism specific to corticotrophs. Given that, in some pools of cells, > 30% of cells made up those in the eGFP ‘high’ peak, it is hard to reconcile that these cells were all made up from melanotrophs of the anterior pituitary. However, we cannot discount the fact that the population may have included some melanotrophs. Second, corticotroph cells can exist in two ‘states’. To date single cell RNA‐seq from pituitaries suggests a spectrum of gene expression (as assessed by levels of *Pomc* transcript) in cells identified as corticotrophs.^39^ Although these studies ‘snapshot’ mRNA levels, variable transcription rates across a population are reminiscent of those described from live cell imaging of pituitary lactotrophs.^40^ Thus, a heterogenous population of corticotroph cells may have been shifted ‘left’ to form a more homogenous population by treatment with DEX.

Because previous *in vitro* studies had shown a persistent stimulation of *Fkbp5* mRNA expression following withdrawal of DEX,^16^ we aimed to determine whether the chronic DEX treatment in mice resulted in sustained transcriptional changes in the corticotrophs that might explain the delay in recovery of the HPA axis. Only one gene was supressed by DEX and exhibited ongoing suppression either 1 or 4 weeks following treatment withdrawal. Two of the 101 genes up‐regulated by DEX remained elevated 4 weeks after withdrawal of the DEX, with five genes showing ongoing up‐regulation 1 week after DEX withdrawal. We thus found little evidence to support persistent global dysregulation of gene expression because of DEX exposure.

We identified several genes with differential expression 1 week after withdrawal of DEX that may play a role in regulation of corticotroph activity. Two G‐protein‐coupled receptors with known significance in corticotroph biology were reduced by DEX exposure but showed significant increase during the recovery process. *Sstr2*, the somatostatin 2 receptor is a target of pasireotide, which is being trialled for use in treatment of Cushing's disease. The reduced expression of *Sstr2* as a result of GC exposure could have implications for the efficacy of this treatment, as well as the selection of patients most likely to benefit.^41^ *Aplr*, the apelin receptor is of interest because apelin is a stimulator of ACTH release and steroidogenesis,^42^, ^43^ and these data suggest a direct role within the pituitary. The transporter *Slc12a5*, (also known as KCC2) was also up‐regulated by DEX; the transporter is inhibited by loop diuretics, and in vitro has been shown to inhibit ACTH release.^44^ Further transporter/ion channel proteins were identified with hitherto unknown function in corticotrophs. We also identified a collection of kallikrein genes up‐regulated by DEX. Previous experiments have shown small reductions in ACTH release in response to hypoglycaemia where rats were co‐treated with a kallikrein inhibitor^45^; thus, this is a potential mechanism further inhibiting ACTH release. A few genes associated with extra‐cellular matrix were identified: *Fbn1*, *Col3a1*, and *Mfap4*. Given persistent changes seen in some of these genes following DEX withdrawal, it is interesting to postulate that DEX has an persistent effect on the intercellular network of corticotrophs^46^; an alteration of pituitary networks that persists after challenge has been described for lactotrophs following first lactation.^47^

There are several limitations to the present study. The number of timepoints we have studied has been limited by feasibility, and thus we have only looked at a small number of ‘snap shots’ during the recovery process. The dynamics of recovery between these time points, and whether these exhibit inter‐individual differences, would be important foci for future work. Furthermore, because we required to pool pituitaries to obtain enough corticotrophs for deep RNA‐seq, our data lack resolution at the individual animal level. We chose this strategy to facilitate the analysis of genes with low expression (e.g., ion channels). Mice likely experienced stress at time of sacrifice and, although procedures were kept consistent for all experiments, perceived stress could account for some of the variation in hormone (especially ACTH) measurements.

To conclude, we established a model of chronic glucocorticoid treatment in mice and found that 4 weeks of DEX exposure had suppressed HPA activity, which persisted 1 week following treatment withdrawal. DEX treatment had a persistent effect on adrenal steroidogenesis and abundance of adrenal transcripts for steroidogenic enzymes that lasted at least 1 week. DEX suppressed corticotroph *Pomc* transcription, an effect that had recovered in some animals 1 week following withdrawal but persisted in others, mirroring the pattern seen in ACTH measurement. Earlier time points during recovery and an increase in the number of individuals would be useful for future experiments examining regulation of suppression, as well as recovery of hypothalamic and corticotroph activity following chronic GC exposure. A persistent but small change in the transcriptome of *Pomc‐*expressing isolated GFP positive cells was identified 4 weeks following DEX withdrawal. This was not associated with altered corticosterone or ACTH production, but might affect further pituitary response to other stimuli (e.g., repeat steroid prescription or stress) and could contribute to the longevity of HPA axis suppression seen in some humans following withdrawal of chronic GC treatment.

## AUTHOR CONTRIBUTIONS

**Peter Duncan:** Investigation; methodology; writing – review and editing. **Heather McClafferty:** Investigation; supervision. **Oscar Nolan:** Investigation. **Qianhui Ding:** Formal analysis; investigation. **Natalie Homer:** Investigation; methodology; writing – review and editing. **Paul Le Tissier:** Writing – original draft. **Brian Walker:** Conceptualization; writing – original draft. **Michael Shipston:** Conceptualization; writing – review and editing. **Nicola Romanò:** Conceptualization; investigation; methodology; writing – review and editing. **Thomas James Gray Chambers:** Conceptualization; formal analysis; funding acquisition; investigation; methodology; writing – original draft.

## CONFLICTS OF INTEREST

The authors declare that no conflicts of interest. TJGC has received a speaker honorarium from Astellas Pharma Inc.

### PEER REVIEW

The peer review history for this article is available at https://publons.com/publon/10.1111/jne.13165.

## Supporting information


**Figure S1.** Assessment of RT‐qPCR products. For this, 10 μL of RT‐qPCR product was run on a 3% agarose gel against a 1‐kB ladder. (A) Pituitary and hypothalamus. (B) Adrenal. All products were of the expected size in all tissues assessed and there was no evidence of primer dimerisation.

**Figure S2.** (A) Bodyweight. DEX inhibited weight gain, but this returned to control levels 4 weeks after stopping treatment. The weight of each mouse in Experiment 2 is shown as representative. Mice were weighed twice a week. Mean and 95% confidence intervals are presented as lines (*n* = 16 cages of three mice). ###*p* < 0.0001 for interaction between weight and time assessed by linear mixed model with cage and individual as random factor to account for repeated measures. Coloured bars at the bottom indicate the time when mice were exposed to DEX. (B) Adrenal weight weights of adrenals from Experiment 3 were not significantly affected by treatment but were reduced and remained lower 4 weeks after stopping DEX. *n* = 6. (C) Dexamethasone levels. Plasma dexamethasone was measured at the end of the rest period (Experiment 2). DEX increased measured DEX, which were undetectable in control groups or following 1 week of withdrawal of DEX (*n* = 6). (D) Comparison of RNA‐seq and RT‐qPCR from whole pituitary. RT‐qPCR of whole pituitary and six key genes for corticotroph function is shown in the top panel. Pituitaries were collected in Experiment 1 (*n* = 5–6 from three cages). Data analysed by linear mixed model with group as dependent variable and cage as random factor. Tukey‐adjusted post‐hoc tests compared to control group are indicated above bars (small asterixis) where significant differences were identified. *** *p* < 0.001, ** *p* < 0.01, * *p* < 0.05. The bottom panel shows for comparison the relative cpm to group A (control) from the RNA‐seq (Experiment 3) (*n* = 3 pools of two or three pituitaries). *** *p* < 0.0001; FDR. Grey dots and boxes represent control mice (group A); yellow dots and boxes represent those who have had 4 weeks of DEX treatment (group B); blue dots and boxes represent those 1 week after withdrawal of DEX (group C); and green dots and boxes represent those 4 weeks after treatment withdrawal (group D). Data analysed by linear mixed model with group as dependent variable and cage as random factor.

**Figure S3.** (A) Fluorescence of isolated cells from each sort. FACS GFP distribution is shown for each sort of two or three anterior pituitaries following treatment (experiment 3). (B) Raw counts of Pax7 and Pcsk2, markers of melanotrophs for comparison with (A). Expression of melanotroph specific genes does not associate with sorts with larger secondary peaks of higher fluorescence. (C) Number of cells, percent of fluorescent cells, and number of reads obtained from each sample from dissociated anterior pituitaries obtained in experiment 3. There was no significant difference between groups (*n* = 3 pools of two or three pituitaries). Grey dots and boxes represent control mice (group A); yellow dots and boxes represent those who have had 4 weeks of DEX treatment (group B); blue dots and boxes represent those one week after withdrawal of DEX (group C); and green dots and boxes represent those 4 weeks after treatment withdrawal (group D).

**Figure S4.** Differential gene expression in FACS‐isolated corticotrophs following DEX treatment and withdrawal. Volcano plots showing differential gene expression from RNA‐seq of FACS‐isolated corticotrophs. (A) Control vs. 4 weeks DEX (group B) exposure. (B) Control vs. 1 week recovery (group C). (C) Control vs. 4 weeks recovery (group D). Yellow points indicate differentially expressed genes FDR <0.05. Genes are labelled where log FC is >|2|.

**Table S1.** (A) Breakdown of hormone measurements for control mice in Experiment 1, which have been grouped together to form group A for ease of comparison with Experiment 2. There was no significant difference between the three time points in the control mice assessed by linear mixed model with treatment and time as dependent variables; Tukey's HSD post‐hoc test was > 0.5 assessing for a difference between each control time point. Data from Experiment 1. (B) Breakdown of hormone assays from Experiment 2 (waking). Mice underwent 30 min of restraint stress (group 1) or not (control group 0) prior to sacrifice which did not affect measured hormones. Significance was assessed by mixed model with cage as random factor and stress and group as exploratory variables. An interaction term was also included which was > 0.05 for each measured hormone. Data from Experiment 2.
**Table S2.** Primers used in qRT‐PCR assays
**Table S3.** Gene ontology analysis of GC induced genes (molecular function)
**Table S4.** Gene ontology analysis of GC suppressed genes (molecular function)

## Data Availability

RNA‐seq data have been deposited in GEO (accession GSE165026). Data are available by request from the corresponding author. The data that support the findings of this study are available from the corresponding author upon reasonable request.
