# Supplementary figures and images for: Corticotroph isolation from Pomc‐eGFP mice reveals sustained transcriptional dysregulation characterising a mouse model of glucocorticoid‐induced suppression of the hypothalamus–pituitary–adrenal axis
Source: J Neuroendocrinol. 2022 Jul 14;34(7):e13165. doi: 10.1111/jne.13165 (PMC9539609; doi:10.1111/jne.13165)

A

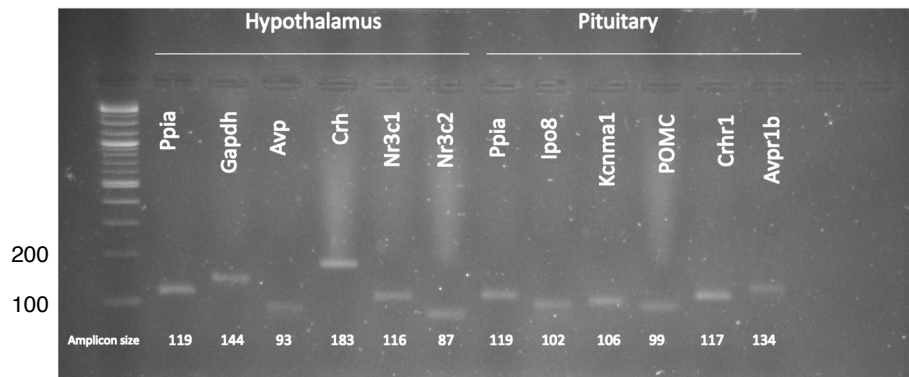

B

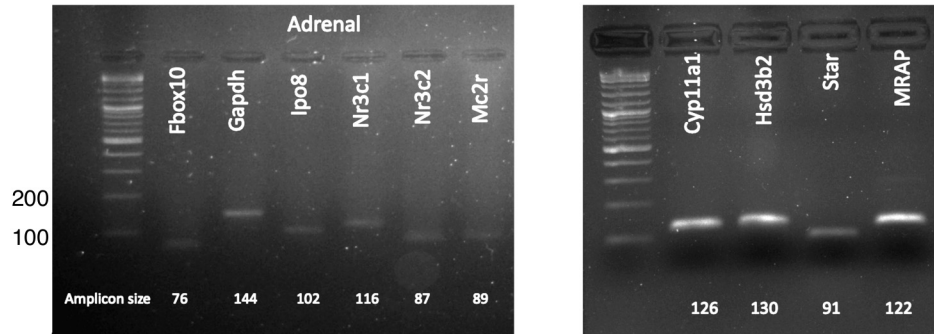

Supplement: Supplementary file 1 — Figure S1. Assessment of RT‐qPCR products. For this, 10 μL of RT‐qPCR product was run on a 3% agarose gel against a 1‐kB ladder. (A) Pituitary and hypothalamus. (B) Adrenal. All products were of the expected size in all tissues assessed and there was no evidence of primer dimerisation. [file JNE-34-e13165-s003.pdf]

**A**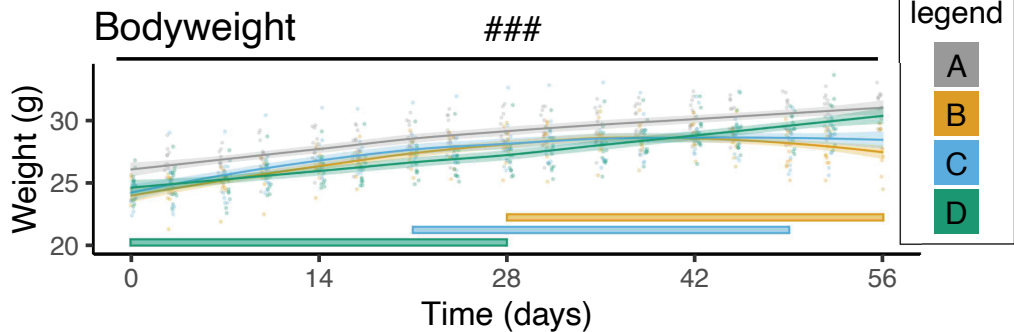**B** Adrenal weight am exp. 3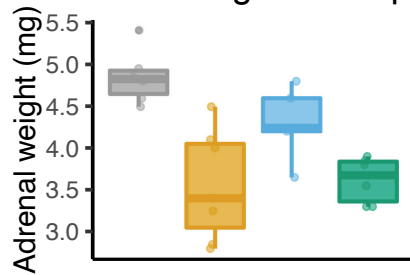**C**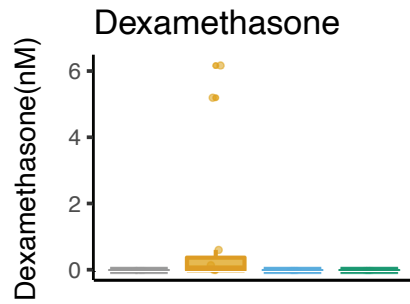**D**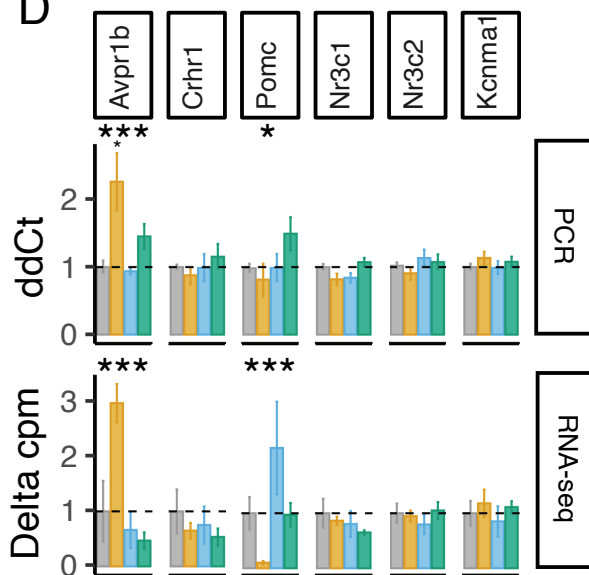

Supplement: Supplementary file 2 — Figure S2. (A) Bodyweight. DEX inhibited weight gain, but this returned to control levels 4 weeks after stopping treatment. The weight of each mouse in Experiment 2 is shown as representative. Mice were weighed twice a week. Mean and 95% confidence intervals are presented as lines (n = 16 cages of three mice). ###p < 0.0001 for interaction between weight and time assessed by linear mixed model with cage and individual as random factor to account for repeated measures. Coloured bars at the bottom indicate the time when mice were exposed to DEX. (B) Adrenal weight weights of adrenals from Experiment 3 were not significantly affected by treatment but were reduced and remained lower 4 weeks after stopping DEX. n = 6. (C) Dexamethasone levels. Plasma dexamethasone was measured at the end of the rest period (Experiment 2). DEX increased measured DEX, which were undetectable in control groups or following 1 week of withdrawal of DEX (n = 6). (D) Comparison of RNA‐seq and RT‐qPCR from whole pituitary. RT‐qPCR of whole pituitary and six key genes for corticotroph function is shown in the top panel. Pituitaries were collected in Experiment 1 (n = 5–6 from three cages). Data analysed by linear mixed model with group as dependent variable and cage as random factor. Tukey‐adjusted post‐hoc tests compared to control group are indicated above bars (small asterixis) where significant differences were identified. *** p < 0.001, ** p < 0.01, * p < 0.05. The bottom panel shows for comparison the relative cpm to group A (control) from the RNA‐seq (Experiment 3) (n = 3 pools of two or three pituitaries). *** p < 0.0001; FDR. Grey dots and boxes represent control mice (group A); yellow dots and boxes represent those who have had 4 weeks of DEX treatment (group B); blue dots and boxes represent those 1 week after withdrawal of DEX (group C); and green dots and boxes represent those 4 weeks after treatment withdrawal (group D). Data analysed by linear mixed model with group as dependent va [file JNE-34-e13165-s002.pdf]

A

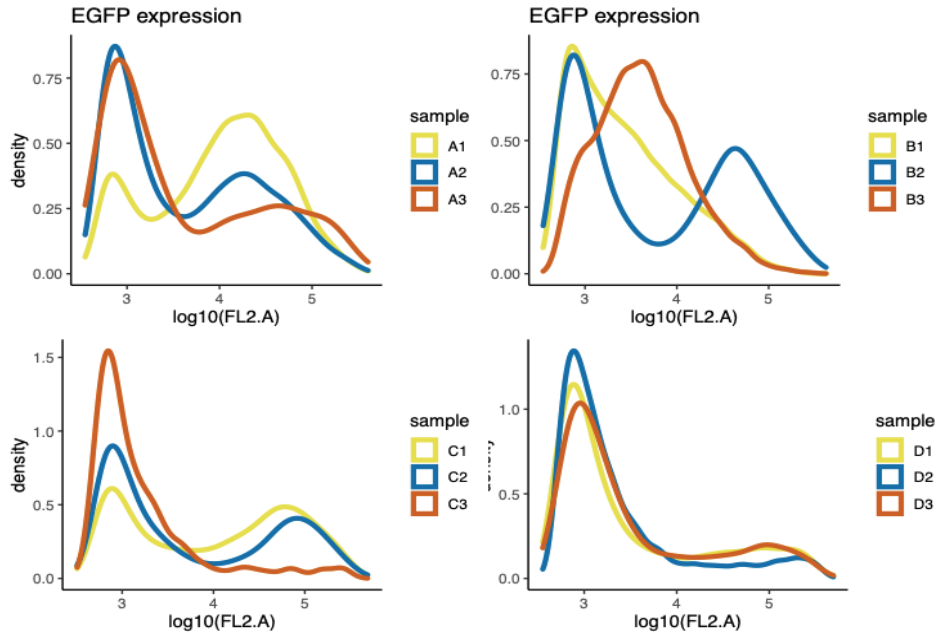

B

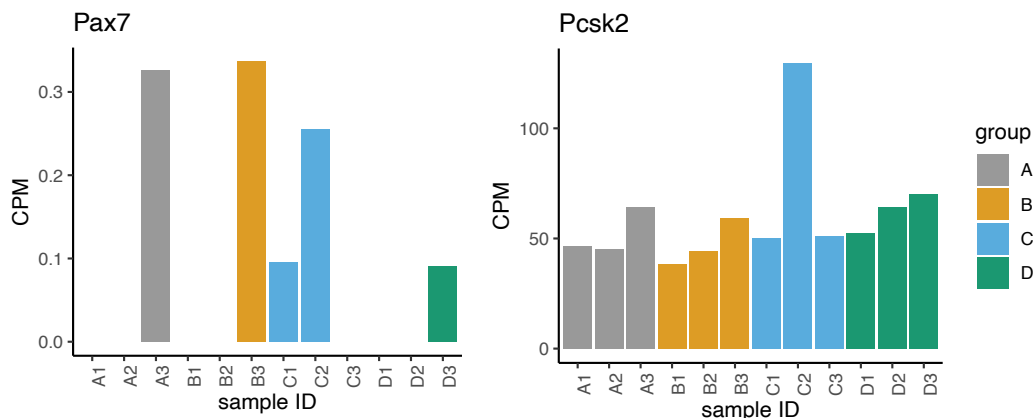

C

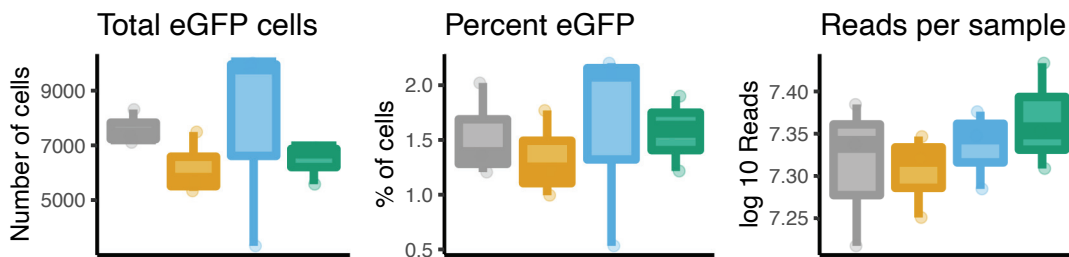

Supplement: Supplementary file 3 — Figure S3. (A) Fluorescence of isolated cells from each sort. FACS GFP distribution is shown for each sort of two or three anterior pituitaries following treatment (experiment 3). (B) Raw counts of Pax7 and Pcsk2, markers of melanotrophs for comparison with (A). Expression of melanotroph specific genes does not associate with sorts with larger secondary peaks of higher fluorescence. (C) Number of cells, percent of fluorescent cells, and number of reads obtained from each sample from dissociated anterior pituitaries obtained in experiment 3. There was no significant difference between groups (n = 3 pools of two or three pituitaries). Grey dots and boxes represent control mice (group A); yellow dots and boxes represent those who have had 4 weeks of DEX treatment (group B); blue dots and boxes represent those one week after withdrawal of DEX (group C); and green dots and boxes represent those 4 weeks after treatment withdrawal (group D). [file JNE-34-e13165-s004.pdf]
