# Supplementary material for: Corticotroph isolation from Pomc‐eGFP mice reveals sustained transcriptional dysregulation characterising a mouse model of glucocorticoid‐induced suppression of the hypothalamus–pituitary–adrenal axis
Source: J Neuroendocrinol. 2022 Jul 14;34(7):e13165. doi: 10.1111/jne.13165 (PMC9539609; doi:10.1111/jne.13165)
Supplement: Supplementary file 5 — Table S1. (A) Breakdown of hormone measurements for control mice in Experiment 1, which have been grouped together to form group A for ease of comparison with Experiment 2. There was no significant difference between the three time points in the control mice assessed by linear mixed model with treatment and time as dependent variables; Tukey's HSD post‐hoc test was > 0.5 assessing for a difference between each control time point. Data from Experiment 1. (B) Breakdown of hormone assays from Experiment 2 (waking). Mice underwent 30 min of restraint stress (group 1) or not (control group 0) prior to sacrifice which did not affect measured hormones. Significance was assessed by mixed model with cage as random factor and stress and group as exploratory variables. An interaction term was also included which was > 0.05 for each measured hormone. Data from Experiment 2. Table S2. Primers used in qRT‐PCR assays Table S3. Gene ontology analysis of GC induced genes (molecular function) Table S4. Gene ontology analysis of GC suppressed genes (molecular function) [file JNE-34-e13165-s005.docx]

**Supplemental table 1A**

| Time.Treatment | **0.CTL (N=6)** | **1.CTL (N=6)** | **4.CTL (N=6)** | **0.DEX (N=6)** | **1.DEX (N=6)** | **4.DEX (N=6)** | **P DEX** | **p time** | **P interaction** |
| --- | --- | --- | --- | --- | --- | --- | --- | --- | --- |
| 11- deoxycorticosterone (nM) |  |  |  |  |  |  | <0.01 | <0.01 | <0.01 |
| Mean (SD) | 1.06 (0.45) | 1.83 (1.16) | 1.23 (0.29) | **0.11** (0.03) | 1.34 (0.61) | 1.68 (0.99) |  |  |  |
| Corticosterone (nM) |  |  |  |  |  |  | <0.01 | <0.01 | <0.01 |
| Mean (SD) | 58.69 (52.78) | 103.19 (110.96) | 35.90 (27.62) | **2.86** (4.33) | 59.36 (31.49) | 95.59 (98.76) |  |  |  |

**Supplemental table 1B**

| Stress.Group | **0.A(N=6)** | **1.A(N=6)** | **0.B (N=6)** | **1.B (N=6)** | **0.C (N=6)** | **1.C (N=6)** | **0.D (N=6)** | **1.D(N=6)** | **P Group** | **P Stress** |
| --- | --- | --- | --- | --- | --- | --- | --- | --- | --- | --- |
| 11-dehydrocorticosterone (nM) |  |  |  |  |  |  |  |  | <0.05 | 0.9 |
| Mean (SD) | 7.68 (2.92) | 8.14 (4.63) | 0.07 (0.04) | 0.75 (0.80) | 7.22 (2.60) | 4.79 (1.60) | 4.13 (1.70) | 11.71 (6.37) |  |  |
| Corticosterone (nM) |  |  |  |  |  |  |  |  | <0.01 | 0.3 |
| Mean (SD) | 567.58 (66.09) | 681.18 (151.55) | 1.60 (1.15) | 65.42 (63.58) | 387.82 (147.15) | 301.94 (72.52) | 493.44 (82.90) | 789.50 (229.82) |  |  |
| ACTH pg/mL |  |  |  |  |  |  |  |  | 0.85 | 0.93 |
| Mean (SD) | 109.66 (57.05) | 107.11 (107.86) | 35.35 (27.92) | 89.52 (129.49) | 102.36 (128.40) | 252.52 (146.64) | 84.62 (90.22) | 62.29 (103.16) |  |  |

**Supplemental Table 2** Primers used in qRT-PCR assays

| Quantitect assays |  |  |
| --- | --- | --- |
| Gene | Assay | Geneglobe ID |
| *Kdm2b* | Mm_Kdm2b_2_SG | QT01044365 |
| *Ppia* | Mm_Ppia_1_SG | QT00247709 |
| *Gapdh* | Mm_Gapdh_3_SG | QT01658692 |
| *Ipo8* | Mm_Ipo8_2_SG | QT01057518 |
| *Avp* | Mm_Avp_1_SG | QT00249389 |
| *Crh* | Mm_Crh_2_SG | QT01055789 |
| *Nr3c1* | Mm_Nr3c1_2_SG | QT01757735 |
| *Nr3c2* | Mm_Nr3c2_2_SG | QT01061333 |
| *Mc2r* | Mm_Mc2r_2_SG | QT01066338 |
| *Cyp11a1* | Mm_Cyp11a1_1_SG | QT00161091 |
| *Hsd3b2* | Mm_Hsd3b2_1_SG | QT01039108 |
| *Pomc* | Mm_Pomc_1_SG | QT00162218 |
| *Crhr1* | Mm_Crhr1_1_SG | QT00106232 |
| *Avpr1b* | Mm_Avpr1b_2_SG | QT01753941 |
| Synthesised primers |  |  |
| Gene | FWD | REV |
| *Star* | TGTCTCGCTCGGGGTCACACA | AGGCAGGGGCACCTCAAGCT |
| *Mrap* | CTGTCCCGCTCACCAGCTAT | GGTAGCCAGGCTCAACCACA |
| *Kcnma1* | GTCTCCAATGAAATGTACACAGAATATC | CTATCATCAGGAGCTTAAGCTTCACA |

**Supplemental Table 3**: Gene ontology analysis of GC induced genes (Molecular function)

| **Enrichment FDR** | **Genes in list** | **Total genes** | **Functional Category** | **Genes** |
| --- | --- | --- | --- | --- |
| **0.000** | 7 | 135 | Hydrolase activity, acting on carbon-nitrogen (but not peptide) bonds | *Mthfd2 Klk1b16 Klk1b26 Klk1b24 Klk1b5 Klk1b4 Klk1b21* |
| **0.000** | 6 | 83 | Hydrolase activity, acting on carbon-nitrogen (but not peptide) bonds, in linear amides | *Klk1b16 Klk1b26 Klk1b24 Klk1b5 Klk1b4 Klk1b21* |
| **0.001** | 7 | 198 | Serine-type endopeptidase activity | *Klk1b5 Prss57 Klk1b16 Klk1b26 Klk1b24 Klk1b4 Klk1b21* |
| **0.002** | 7 | 217 | Serine-type peptidase activity | *Klk1b5 Prss57 Klk1b16 Klk1b26 Klk1b24 Klk1b4 Klk1b21* |
| **0.002** | 7 | 222 | Serine hydrolase activity | *Klk1b5 Prss57 Klk1b16 Klk1b26 Klk1b24 Klk1b4 Klk1b21* |
| **0.003** | 3 | 23 | Structural constituent of muscle | *Myom1 Myh11 Krt19* |
| **0.004** | 9 | 469 | Endopeptidase activity | *Atg4a Klk1b5 Prss57 Klk1b16 Klk1b26 Adamts14 Klk1b24 Klk1b4 Klk1b21* |
| **0.004** | 11 | 683 | Peptidase activity | *Atg4a Klk1b26 Adamts14 Klk1b5 Klk1b21 Prss57 Usp54 Klk1b16 Klk1b24 Klk1b4 Adamtsl3* |
| **0.006** | 2 | 7 | Netrin receptor activity | *Unc5b Unc5a* |
| **0.009** | 8 | 427 | Metal ion transmembrane transporter activity | *Atp7b Slc12a5 Atp2a1 Kcnh4 Slc24a4 Slc5a3 Asic2 Tmem37* |
| **0.009** | 10 | 654 | Peptidase activity, acting on L-amino acid peptides | *Atg4a Klk1b5 Prss57 Usp54 Klk1b16 Klk1b26 Adamts14 Klk1b24 Klk1b4 Klk1b21* |
| **0.014** | 2 | 12 | Diacylglycerol kinase activity | *Dgki Dgkk* |
| **0.020** | 9 | 622 | Cation transmembrane transporter activity | *Atp7b Slc12a5 Atp2a1 Kcnh4 Slc24a4 Slc5a3 Slc44a4 Asic2 Tmem37* |
| **0.030** | 2 | 19 | NAD+ kinase activity | *Dgki Dgkk* |
| **0.038** | 8 | 574 | Inorganic cation transmembrane transporter activity | *Atp7b Slc12a5 Atp2a1 Kcnh4 Slc24a4 Slc5a3 Asic2 Tmem37* |
| **0.040** | 10 | 851 | Ion transmembrane transporter activity | *Atp7b Slc12a5 Atp2a1 Kcnh4 Slc7a5 Slc24a4 Slc5a3 Slc44a4 Asic2 Tmem37* |
| **0.045** | 6 | 362 | Monovalent inorganic cation transmembrane transporter activity | *Slc12a5 Atp2a1 Kcnh4 Slc24a4 Slc5a3 Asic2* |

**Supplemental Table** 4: Gene ontology analysis of GC suppressed genes (Molecular function)

| **Enrichment FDR** | **Genes in list** | **Total genes** | **Functional Category** | **Genes** |
| --- | --- | --- | --- | --- |
| **0.001** | 3 | 51 | Antigen binding | *H2-Q6 H2-Aa H2-Ab1* |
| **0.001** | 5 | 291 | Peptide binding | *Sstr2 H2-Q6 H2-Aa H2-Ab1 Cd74* |
| **0.001** | 3 | 33 | Peptide antigen binding | *H2-Q6 H2-Aa H2-Ab1* |
| **0.001** | 2 | 7 | CD4 receptor binding | *Il16 Cd74* |
| **0.001** | 5 | 358 | Amide binding | *Sstr2 H2-Q6 H2-Aa H2-Ab1 Cd74* |
| **0.001** | 2 | 12 | Platelet-derived growth factor binding | *Pdgfrb Col3a1* |
| **0.002** | 8 | 1443 | Protein dimerization activity | *Rrm2 Pecam1 Top2a Itgal Adgrl4 H2-Aa H2-Q6 H2-Ab1* |
| **0.005** | 8 | 1628 | Signaling receptor binding | *Pomc H2-Q6 Il16 Cd74 Pdgfrb Col3a1 Kdr Nmb* |
| **0.005** | 3 | 144 | Growth factor binding | *Pdgfrb Col3a1 Kdr* |
| **0.005** | 5 | 607 | Protein heterodimerization activity | *Top2a Itgal H2-Aa H2-Q6 H2-Ab1* |
| **0.005** | 2 | 34 | Neuropeptide receptor binding | *Pomc Nmb* |
| **0.013** | 3 | 223 | Cell adhesion molecule binding | *Col3a1 Itgal Kdr* |
| **0.014** | 2 | 68 | Transmembrane receptor protein tyrosine kinase activity | *Pdgfrb Kdr* |
| **0.014** | 6 | 1207 | Protein-containing complex binding | *Nckap1l Cd74 Col3a1 Itgal Kdr H2-Q6* |
| **0.020** | 2 | 85 | Transmembrane receptor protein kinase activity | *Pdgfrb Kdr* |
| **0.023** | 8 | 2327 | Enzyme binding | *Nckap1l Ccnb2 Top2a Pdgfrb Col3a1 Cd74 H2-Ab1 Pecam1* |
| **0.026** | 4 | 633 | Protein kinase activity | *Ccnb2 Pbk Pdgfrb Kdr* |
| **0.029** | 8 | 2473 | Signaling receptor activity | *Adgrl4 Aplnr Sstr2 Cd74 Pdgfrb Itgal Rtn4rl1 Kdr* |
| **0.030** | 2 | 118 | Integrin binding | *Col3a1 Kdr* |
| **0.030** | 8 | 2521 | Molecular transducer activity | *Adgrl4 Aplnr Sstr2 Cd74 Pdgfrb Itgal Rtn4rl1 Kdr* |
| **0.031** | 2 | 128 | Extracellular matrix structural constituent | *Col3a1 Mfap4* |
| **0.032** | 2 | 136 | Protein tyrosine kinase activity | *Pdgfrb Kdr* |
| **0.032** | 4 | 735 | Phosphotransferase activity, alcohol group as acceptor | *Ccnb2 Pbk Pdgfrb Kdr* |
| **0.032** | 2 | 140 | G protein-coupled peptide receptor activity | *Aplnr Sstr2* |
| **0.035** | 2 | 149 | Peptide receptor activity | *Aplnr Sstr2* |
| **0.039** | 2 | 162 | Protein kinase regulator activity | *Ccnb2 Nckap1l* |
| **0.043** | 4 | 840 | Kinase activity | *Ccnb2 Pbk Pdgfrb Kdr* |
| **0.044** | 7 | 2304 | Transmembrane signaling receptor activity | *Adgrl4 Aplnr Sstr2 Cd74 Pdgfrb Itgal Kdr* |
| **0.050** | 2 | 194 | Kinase regulator activity | *Ccnb2 Nckap1l* |
