# Supplementary material for: Corticotroph isolation from Pomc‐eGFP mice reveals sustained transcriptional dysregulation characterising a mouse model of glucocorticoid‐induced suppression of the hypothalamus–pituitary–adrenal axis
Source: J Neuroendocrinol. 2022 Jul 14;34(7):e13165. doi: 10.1111/jne.13165 (PMC9539609; doi:10.1111/jne.13165)
Supplement: Supplementary file 4 — Figure S4. Differential gene expression in FACS‐isolated corticotrophs following DEX treatment and withdrawal. Volcano plots showing differential gene expression from RNA‐seq of FACS‐isolated corticotrophs. (A) Control vs. 4 weeks DEX (group B) exposure. (B) Control vs. 1 week recovery (group C). (C) Control vs. 4 weeks recovery (group D). Yellow points indicate differentially expressed genes FDR <0.05. Genes are labelled where log FC is >|2|. [file JNE-34-e13165-s001.pdf]

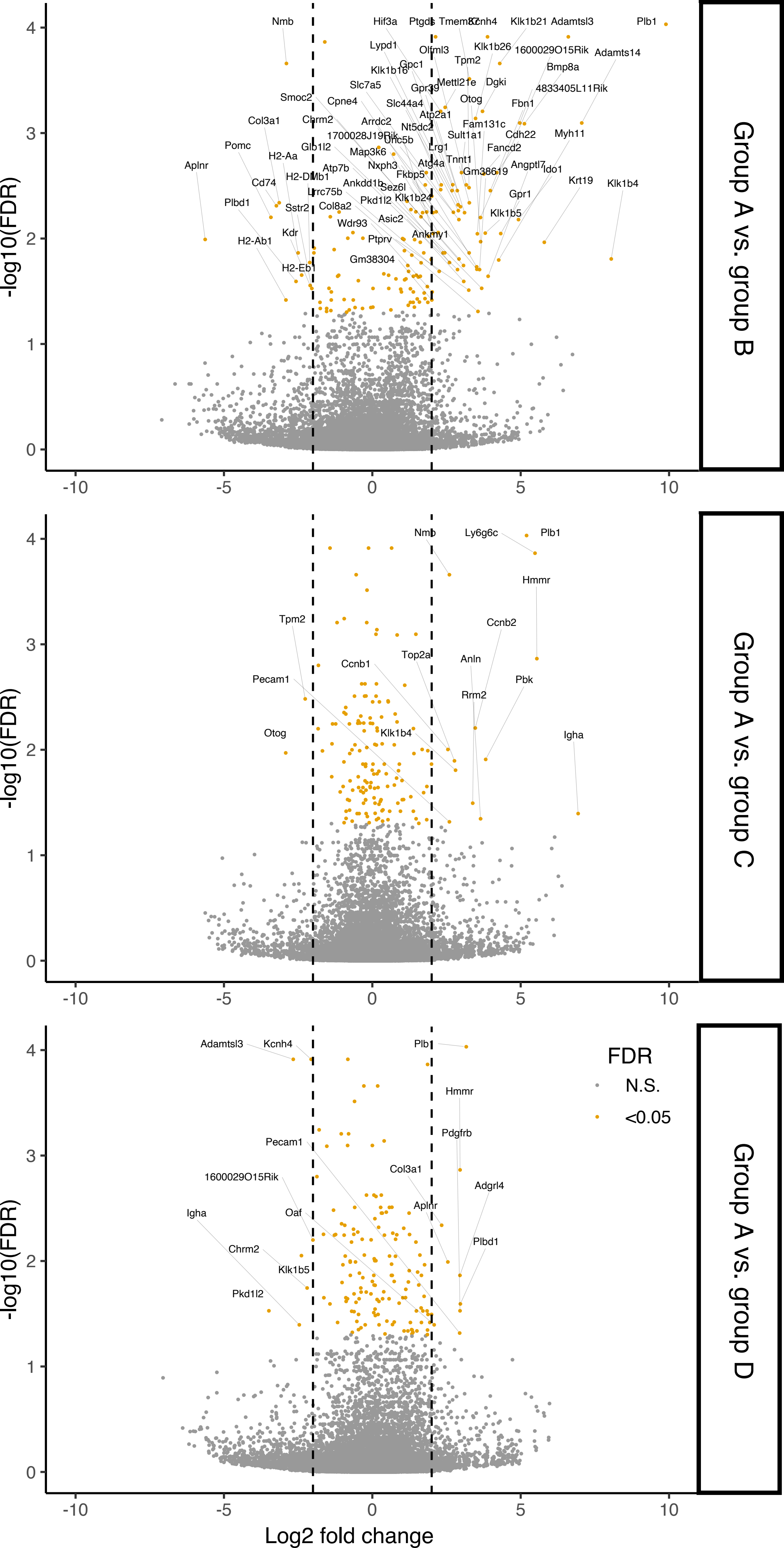
Group A vs. group B
-log10(FDR)
Nmb
Hif3a
Ptgds
Tmem87
Kcnh4
Klk1b21
Adamtsl3
Plb1
Lypd1
Olfml3
Klk1b26
1600029O15Rik
Adamts14
Gpc1
Tpm2
Bmp8a
Klk1b16
Slc7a5
Gpr39
Mettl21e
Dgki
4833405L11Rik
Smoc2
Cpne4
Slc44a4
Otog
Fbn1
Col3a1
Chrm2
Arrdc2
Atp2a1
Fam131c
Nt5dc2
Myh11
1700028J19Rik
Unc5b
Sult1a1
Cdh22
Pomc
Glb1l2
Lrg1
Fancd2
H2-Aa
Map3k6
Tnnt1
Aplnr
Atp7b
Nxph3
Atg4a
Angptl7
Ido1
H2-DMb1
Fkbp5
Gm38619
Cd74
Ankdd1b
Krt19
Klk1b4
Sez6l
Lrrc75b
Gpr1
Plbd1
Pkd1l2
Klk1b24
Sstr2
Col8a2
Klk1b5
Asic2
Wdr93
Kdr
Ankmy1
H2-Ab1
Ptprv
Gm38304
H2-Eb1
Group A vs. group C
-log10(FDR)
Nmb
Ly6g6c
Plb1
Hmmr
Tpm2
Ccnb2
Top2a
Anln
Ccnb1
Pecam1
Pbk
Rrm2
Otog
Klk1b4
Igha
Group A vs. group D
-log10(FDR)
Log2 fold change
Adamtsl3
Kcnh4
Plb1
Hmmr
Pdgfrb
Pecam1
Col3a1
Adgrl4
1600029O15Rik
Aplnr
Igha
Oaf
Plbd1
Chrm2
Klk1b5
Pkd1l2
FDR
N.S.
<0.05
